# The IQD Gene Family in Soybean: Structure, Phylogeny, Evolution and Expression

**DOI:** 10.1371/journal.pone.0110896

**Published:** 2014-10-24

**Authors:** Lin Feng, Zhu Chen, Hui Ma, Xue Chen, Yuan Li, Yiyi Wang, Yan Xiang

**Affiliations:** 1 Laboratory of Modern Biotechnology, School of Forestry and Landscape Architecture, Anhui Agricultural University, Hefei, China; 2 Key Laboratory of Crop Biology of Anhui Agriculture University, Hefei, China; University of Lausanne, Switzerland

## Abstract

Members of the plant-specific IQ67-domain (IQD) protein family are involved in plant development and the basal defense response. Although systematic characterization of this family has been carried out in *Arabidopsis*, tomato (*Solanum lycopersicum*), *Brachypodium distachyon* and rice (*Oryza sativa*), systematic analysis and expression profiling of this gene family in soybean (*Glycine max*) have not previously been reported. In this study, we identified and structurally characterized IQD genes in the soybean genome. A complete set of 67 soybean IQD genes (*GmIQD1*–*67*) was identified using Blast search tools, and the genes were clustered into four subfamilies (IQD I–IV) based on phylogeny. These soybean IQD genes are distributed unevenly across all 20 chromosomes, with 30 segmental duplication events, suggesting that segmental duplication has played a major role in the expansion of the soybean IQD gene family. Analysis of the Ka/Ks ratios showed that the duplicated genes of the GmIQD family primarily underwent purifying selection. Microsynteny was detected in most pairs: genes in clade 1–3 might be present in genome regions that were inverted, expanded or contracted after the divergence; most gene pairs in clade 4 showed high conservation with little rearrangement among these gene-residing regions. Of the soybean IQD genes examined, six were most highly expressed in young leaves, six in flowers, one in roots and two in nodules. Our qRT-PCR analysis of 24 soybean IQD III genes confirmed that these genes are regulated by MeJA stress. Our findings present a comprehensive overview of the soybean IQD gene family and provide insights into the evolution of this family. In addition, this work lays a solid foundation for further experiments aimed at determining the biological functions of soybean IQD genes in growth and development.

## Introduction

Ca^2 +^ is a pivotal cytosolic second messenger involved in many physiological processes such as plant growth [1], plant-pathogen interactions [2], photosynthetic electron transport and photophosphorylation [3], regulation of stomatal aperture [4], hormonal regulation [5] and so on. Plants produce calcium signals by adjusting cytoplasm Ca^2+^ levels at specific times, places and concentrations [6], responding to numerous extracellular stimuli including physical signals (light, temperature, gravity, etc.) and chemical signals (plant hormones, pathogenic bacteria inducing factors, etc.) [7].

The transmission of these intracellular calcium signals relies on the oscillation signal generated by voltage- and ligand-gated Ca^2+^-permeable channels (influx) and by Ca^2+^-ATPases and antiporters (efflux) to return to resting Ca^2+^ levels [8], [9]. The conduction of calcium signals is also dependent on downstream Ca^2+^ sensors. These Ca^2+^ sensors detect changes in Ca^2+^ levels by binding to Ca^2+^ via domains such as EF hands, which undergo conformational changes [10]. Consequently, calcium signature information is decoded and relayed by these Ca^2+^ sensors [6], [11]–[13].

To date, approximately four major classes of Ca^2+^ sensors have been identified in plants. Most of these sensors contain the Ca^2+^-binding EF-hand motif, a conserved helix-loop-helix structure that can bind to a single Ca^2+^ ion [7]. The four major classes of Ca^2+^ sensors are as follows: class A: calmodulin (CaM), containing four EF-hand motifs; class B: calcineurin B-like (CBL) proteins, possessing three EF-hand motifs; class C: Ca^2+^-dependent protein kinases (CDPK), containing four EF-hand motifs and a Ca^2+^-dependent Ser/Thr protein kinase domain and class D: lacking EF-hand motifs [7], [14]–[19].

Calmodulin (CaM) and calcineurin B-like (CBL) proteins, which lack catalytic activity, are sometimes referred to as “Ca^2+^ sensor relays” [15], [19], [20]. In contrast, CDPK proteins, which function as catalytic effectors, are referred to as “Ca^2+^ sensor responders” [18]. Among these Ca^2+^-binding proteins, calmodulin is the most extensively studied Ca^2+^ sensor. Calmodulin is small, acid resistant, heat resistant and highly stable. This multifunctional protein is widespread in eukaryotic cells, highly conserved and has at least 30 multiple target proteins or enzymes [21]–[23].

To mediate intracellular calcium signaling pathways, Ca^2+^ sensor relays expose their negative hydrophobic surfaces after they undergo conformational changes induced by Ca^2+^ binding. As a result, the affinity between Ca^2+^ sensor relays and their effectors are enhanced, and the biochemical activities of target proteins are modulated by Ca^2+^ sensor relays [6], [12], [14], [19].

In the final phase of the calcium signal transduction process, the target effectors respond to specific extracellular signals by regulating various cellular activities. Calmodulin interacts with numerous target proteins termed calmodulin-binding proteins (CaMBPs), mainly by recognizing and targeting calmodulin-binding domains (CaMBD; basic amphiphilic helices usually composed of 16–35 amino acid residues) in the CaMBPs via its negative hydrophobic pockets [12], [22], [24].

CaMBD amino acid sequences contain three CaMBD motifs that are grouped into two categories, including a Ca^2+^-independent motif termed the IQ motif and two Ca^2+^-dependent motifs referred to as the l-5-10 motif and the l-8-14 motif. The number and positions of these motifs in different CaMBPs are variable [25]–[27]. Due to the diversity of the motif arrangement, there are a variety of diverse CaMBPs with disparate functions, which are implicated in plant development, metabolic regulation, stress reactions, defense reactions, transcriptional regulation and so on [28], [29].

Plant-specific IQ67 domain (IQD) protein families were first identified in *Arabidopsis* and rice by Abel et al. (2005) [30]. These proteins have two common features in their IQ67 domains (67 conserved amino acid residues) [31]. One feature is 1–3 copies IQ motifs are separated by 11 and 15 residues and overlapped certain regions with 1–4 copies 1-5-10 motif as well as 1-8-14 motif. The other hallmark is a highly conserved exon-intron boundary that interrupts codons 16 and 17 with a 0 phase intron [31]–[33]. To date, IQD gene families have been identified in four genomes (*Arabidopsis*, rice, tomato and *Brachypodium distachyon*), including approximately 30 IQD genes per species (33 in *Arabidopsi*s, 29 in rice, 34 in tomato and 23 in *Brachypodium distachyon*), and the functions of several members of the IQD family have been reported [30], [34], [35]. Overexpression of *Arabidopsis IQD1* can mediate the accumulation of glucosinolate in response to insect herbivory [31]. *Arabidopsis IQD22* contributes to the negative feedback regulation of gibberellin (GA) [36]. The tomato *SUN* gene plays a role in elongating tomato fruit by increasing the vertical division of cells and reducing horizontal cell divisions [37]–[39].

Soybean serves as a major source of vegetable proteins and edible oil and own the ability to fix atmospheric nitrogen via its intimate symbiosis with microorganisms. This crucial leguminous seed crop has high economic and nutritional value [40], [41], serving as a main food crop for humans and animals in many parts of the world. Nevertheless, soybean production is limited by many biotic stresses. For example, Asian soybean rust (ASR, caused by the fungus *Phakopsora pachyrhizi*) results in soybean yield losses ranging from 10 to 80% in various countries [42], [43].

In this study, we identified and characterized 67 soybean IQD genes. Among these, we subjected 24 IQD III genes to qRT-PCR analysis to investigate their response to MeJA stress. We determined that all 24 soybean IQD genes are stress-responsive. Our findings lay the foundation of further investigations of the functions of these calmodulin target proteins in soybean.

## Results

### Identification and annotation of IQD genes in soybean

As described in previous studies, IQD proteins, which are plant-specific calcium-dependent calmodulin-binding proteins, contain 67 amino acid residues in their central regions referred to as the IQ67 domain, including three CaM recruitment motifs exhibiting unique repetitive patterns. The Ca^2+^-independent IQ motif (IQxxxRGxxxR or its more relaxed version [ILV]QxxxRxxxx [R, K]) is present in 1–3 copies and overlaps with 1–4 copies of the Ca^2+^-dependent 1-5-10 motif ([FILVW]x_3_ [FILV]x_4_ [FILVW]) and the 1-8-14 motif ([FILVW]x_6_ [FAILVW]x_5_ [FILVW]) by several conserved basic and hydrophobic amino acid residues flanking these motifs [30], [34]. These features allow the IQ67 domain to fold into a basic amphiphilic helix structure, which enables these proteins to perform specific roles.

To conducted genome-wide identification of IQD gene families in soybean, we performed *Glycine max* genome BLASTP analysis. Through removing redundant sequences and pattern identificating, a total of 67 IQD genes were identified in the soybean genome, which is twice that of *Arabidopsis* (Table 1 and 2). We named these 67 IQD genes *GmIQD1* to *GmIQD67* according to their physical locations (from top to bottom) on chromosomes 1–20 (Table 1).

**Table 1:** List of 67 IQD genes identified in soybean, their sequence characteristics and subcellular localization.

Name	Gene Identifier	Chr.	Location coordinates (5′-3′)	ORF length (bp)	Protein			Exons	subcellular localization	
					Length (aa.)	pI	Mol.Wt. (kD)		WoLF PSORT	TargetP
GmIQD1	Glyma01g01030	1	681417–683646	1263	420	10.3	46.6	3	N	M0.65/4
GmIQD2	Glyma01g05100	1	4750065–4755456	1692	563	9.7	61.7	6	N	C0.68/3
GmIQD3	Glyma01g42620	1	53843322–53846963	1191	396	10.4	44.4	4	N	M0.54/5
GmIQD4	Glyma02g00710	2	502944–506146	1254	417	9.6	46.8	3	N	?
GmIQD5	Glyma02g02370	2	1778568–1785636	1692	563	9.8	61.7	6	N	M0.43/5
GmIQD6	Glyma02g15590	2	14083370–14089609	1608	535	10.8	60.1	5	N	?
GmIQD7	Glyma03g33560	3	41092523–41096935	1434	477	10.0	53.3	5	N	?
GmIQD8	Glyma03g40630	3	46330165–46332185	1125	374	10.5	42.4	3	N	?
GmIQD9	Glyma04g02830	4	2030287–2036251	2715	904	5.4	99.2	6	N	?
GmIQD10	Glyma04g05520	4	4187757–4190317	1353	450	10.5	49.9	5	N	?
GmIQD11	Glyma04g23760	4	27192306–27195532	1353	450	9.8	50.8	5	N	?
GmIQD12	Glyma04g34150	4	40144241–40151603	1752	583	9.4	64.6	6	N	?
GmIQD13	Glyma04g41380	4	47220698–47225472	1392	463	9.6	51.7	4	N	?
GmIQD14	Glyma05g01240	5	785189–792757	1761	586	9.7	64.9	6	N	?
GmIQD15	Glyma05g03450	5	2638386–2641896	1338	445	10.0	48.9	4	N	?
GmIQD16	Glyma05g35920	5	39871246–39873985	1128	375	10.0	41.4	4	N	M0.81/3
GmIQD17	Glyma06g02841	6	1950849–1956820	2532	843	5.7	92.8	6	N	?
GmIQD18	Glyma06g05530	6	3957759–3960421	1353	450	10.7	49.8	5	E.R.	?
GmIQD19	Glyma06g13470	6	10606168–10611219	1341	446	9.7	50.1	4	N	?
GmIQD20	Glyma06g20341	6	16752231–16759304	1755	584	9.5	64.9	6	N	?
GmIQD21	Glyma07g01040	7	607467–610485	1302	433	10.0	47.9	5	N	?
GmIQD22	Glyma07g01760	7	1164144–1167157	1191	396	10.2	44.6	3	N	?
GmIQD23	Glyma07g05680	7	4335391–4339373	1641	546	10.3	61.2	5	N	?
GmIQD24	Glyma07g14910	7	14801071–14803234	1398	465	10.0	51.8	3	C	S0.90/1
GmIQD25	Glyma07g32531	7	37416802–37421879	873	290	10.6	32.8	5	N	?
GmIQD26	Glyma07g32860	7	37753882–37759623	1602	533	10.9	59.7	5	N	?
GmIQD27	Glyma08g03710	8	2630927–2633769	1311	436	10.2	48.2	3	N	M0.77/4
GmIQD28	Glyma08g20430	8	15453660–15456579	1266	421	10.4	46.4	5	N	M0.56/5
GmIQD29	Glyma08g21430	8	16271106–16273575	1209	402	10.3	45.2	3	N	?
GmIQD30	Glyma08g40880	8	40742659–40748073	1644	547	9.8	60.6	6	C	C0.75/3
GmIQD31	Glyma09g26630	9	33163730–33169453	1449	482	10.0	53.3	4	C	M0.51/4
GmIQD32	Glyma09g30780	9	37552192–37557238	1305	434	10.1	48.0	6	N	?
GmIQD33	Glyma09g35920	9	41794962–41798738	1407	468	9.9	52.6	5	N	M0.60/4
GmIQD34	Glyma10g00630	10	386683–389158	1272	423	9.5	47.5	3	N	M0.33/5
GmIQD35	Glyma10g05720	10	4477640–4481520	1425	474	10.0	52.8	5	N	?
GmIQD36	Glyma10g35721	10	43974896–43978361	1452	483	10.6	53.0	5	N	?
GmIQD37	Glyma10g38310	10	46118444–46123432	1395	464	10.4	51.0	4	C	M0.54/5
GmIQD38	Glyma10g39030	10	46764292–46767407	1410	469	9.7	52.0	4	N	?
GmIQD39	Glyma11g20880	11	17714458–17717939	1374	457	10.0	51.7	5	N	M0.67/3
GmIQD40	Glyma12g01410	12	842971–846738	1383	460	10.0	51.8	5	N	M0.56/4
GmIQD41	Glyma12g31610	12	35181013–35188577	1269	422	9.9	46.5	6	N	?
GmIQD42	Glyma12g35711	12	38833825–38837834	885	294	9.8	34.2	5	N	?
GmIQD43	Glyma13g20070	13	23539750–23543840	1413	470	10.1	52.3	5	N	C0.34/5
GmIQD44	Glyma13g24070	13	27399608–27404534	774	257	10.5	29.3	4	N	?
GmIQD45	Glyma13g30590	13	33154582–33158861	900	299	10.4	33.6	5	N	?
GmIQD46	Glyma13g34700	13	36237460–36241896	1173	390	9.8	45.5	6	N	?
GmIQD47	Glyma13g38800	13	39521853–39528595	1278	425	9.9	47.1	6	N	?
GmIQD48	Glyma13g42440	13	42441870–42445047	1239	412	10.3	45.8	5	N	?
GmIQD49	Glyma13g43031	13	42796469–42804226	1143	380	10.2	43.4	3	N	?
GmIQD50	Glyma14g11050	14	9335703–9339095	1254	417	10.3	47.3	5	N	?
GmIQD51	Glyma14g25860	14	31470493–31475301	1377	458	10.0	51.3	4	N	?
GmIQD52	Glyma15g02370	15	1595640–1598698	1137	378	10.2	43.3	3	N	?
GmIQD53	Glyma15g02940	15	2051157–2053854	1251	416	10.3	45.9	5	C	?
GmIQD54	Glyma15g08660	15	6125483–6129362	927	308	10.3	34.7	5	N	?
GmIQD55	Glyma16g02240	16	1759053–1762330	1653	550	10.2	61.6	5	N	M0.37/5
GmIQD56	Glyma16g22935	16	26564269–26565120	426	141	11.1	16.3	2	C	M0.82/4
GmIQD57	Glyma16g32161	16	35337880–35343544	1434	477	10.0	52.8	4	C	M0.56/4
GmIQD58	Glyma17g10660	17	8002515–8009332	1767	588	9.5	65.0	6	N	?
GmIQD59	Glyma17g14000	17	10763173–10767584	1344	447	10.0	48.9	4	N	?
GmIQD60	Glyma17g23770	17	23932487–23938307	1386	461	10.4	50.7	5	N	?
GmIQD61	Glyma17g34520	17	38500561–38503843	1242	413	10.4	46.7	5	N	?
GmIQD62	Glyma18g16130	18	16440695–16446996	1644	547	9.7	60.3	6	N	C0.65/4
GmIQD63	Glyma19g36270	19	43610551–43615073	1434	477	10.0	53.3	5	N	?
GmIQD64	Glyma19g43300	19	48995941–48998264	1113	370	10.6	42.2	3	N	?
GmIQD65	Glyma20g28800	20	37708013–37709907	1434	477	9.8	52.7	3	N	C0.67/5
GmIQD66	Glyma20g29550	20	38392614–38397440	1371	456	10.5	50.3	4	C	?
GmIQD67	Glyma20g31810	20	40423269–40426995	1470	489	10.4	53.7	5	C	?

bp, base pair; aa, amino acids; kD, kilo Dalton.

WoLF PSORT predictions: N (nucleus), C (chloroplast), ER (endoplasmic reticulum).

TargetP predictions: C (chloroplast), M (mitochondrion), S (secretory pathway),? (any other location); values indicate score (0.00–1.00) and reliability class (1–5; best class is 1).

**Table 2:** Number of IQD genes in the soybean, rice, *Arabidopsis*, tomato and *Brachypodium distachyon* genomes.

Subfamily	Soybean	Arabidopsis	Tomato	Rice	Brachypodium distachyon
I	27	13	15	11	9
II	6	4	6	1	2
III	24	10	10	10	9
IV	10	5	3	3	2
Outgroup		1		4	1
Total number	67	33	34	29	23

The physicochemical parameters of each gene were calculated using ExPASy. Although all of the GmIQD genes encode the conserved IQ67 domains (Figure S1), their sequences are highly diverse with respect to size (141–904 aa) and molecular mass (16.3–99.2 kDa; Table 1). Almost all soybean IQD proteins (97%) have relatively high isoelectric points (pI>7.0 with an average of 10.1), except for *GmIQD9* (pI 5.4) and *GmIQD17* (pI 5.7; Table 1). All soybean IQD proteins were submitted to TargetP and Wolf PSORT to predict their subcellular localizations. Wolf PSORT revealed that fifty-seven soybean IQD proteins are localized to the nucleus, nine to the chloroplast and one to the endoplasmic reticulum. TargetP analysis revealed that fifteen soybean IQD proteins are located in the mitochondria, five in the chloroplast, one in the secretory pathway and forty-six in other compartments (Table 1). The detailed parameters are provided in Table 1.

### Phylogenetic and structural analyses of the soybean IQD genes

**Figure 1:**
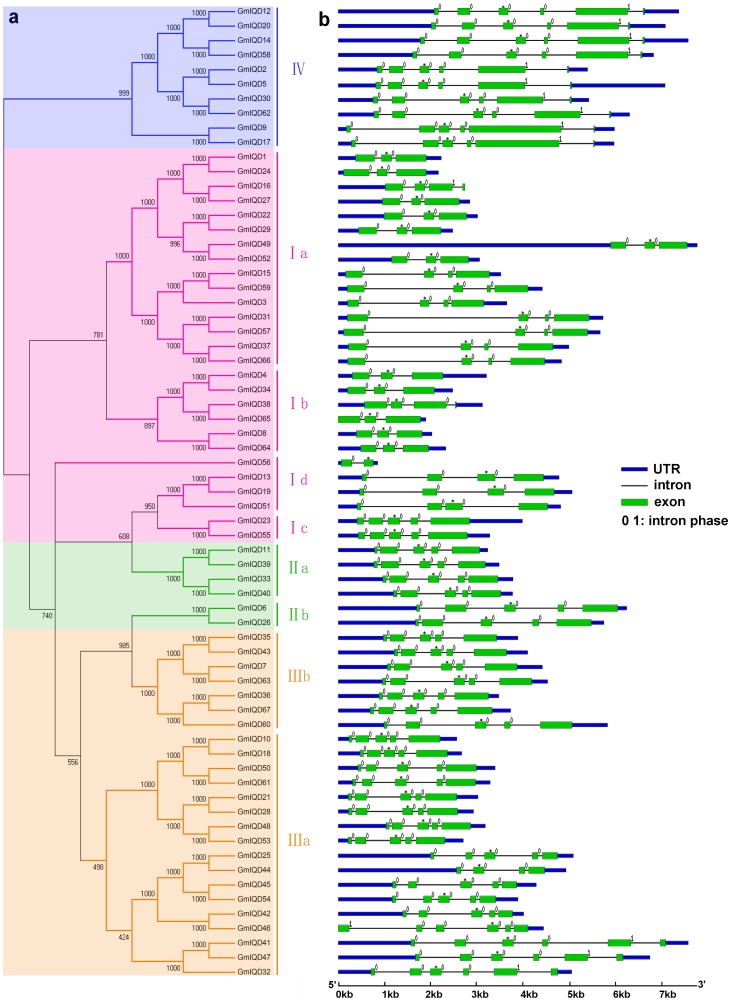
Phylogenetic relationships and exon/intron organization of soybean IQD genes. **a:** Unrooted tree generated with Clustal X2.0 using the full-length amino acid sequences of the 67 soybean IQD proteins by the Neighbor-Joining (NJ) method with 1,000 bootstrap replicates. Subfamilies and subclasses of IQD genes (I–IV) are highlighted with different colored backgrounds and vertical bars next to the gene names of the tree. **b:** Exon/intron organization of soybean IQD genes. Green boxes represent exons and black lines represent introns. Untranslated regions (UTRs) are indicated by blue boxes. Numbers 0 and 1 represent the splicing phases. The sizes of exons and introns can be estimated using the scale at the bottom. The exon encoding amino acids 17–67 of the IQ67 domain in each soybean gene is indicated with an asterisk.

**Figure 2:**
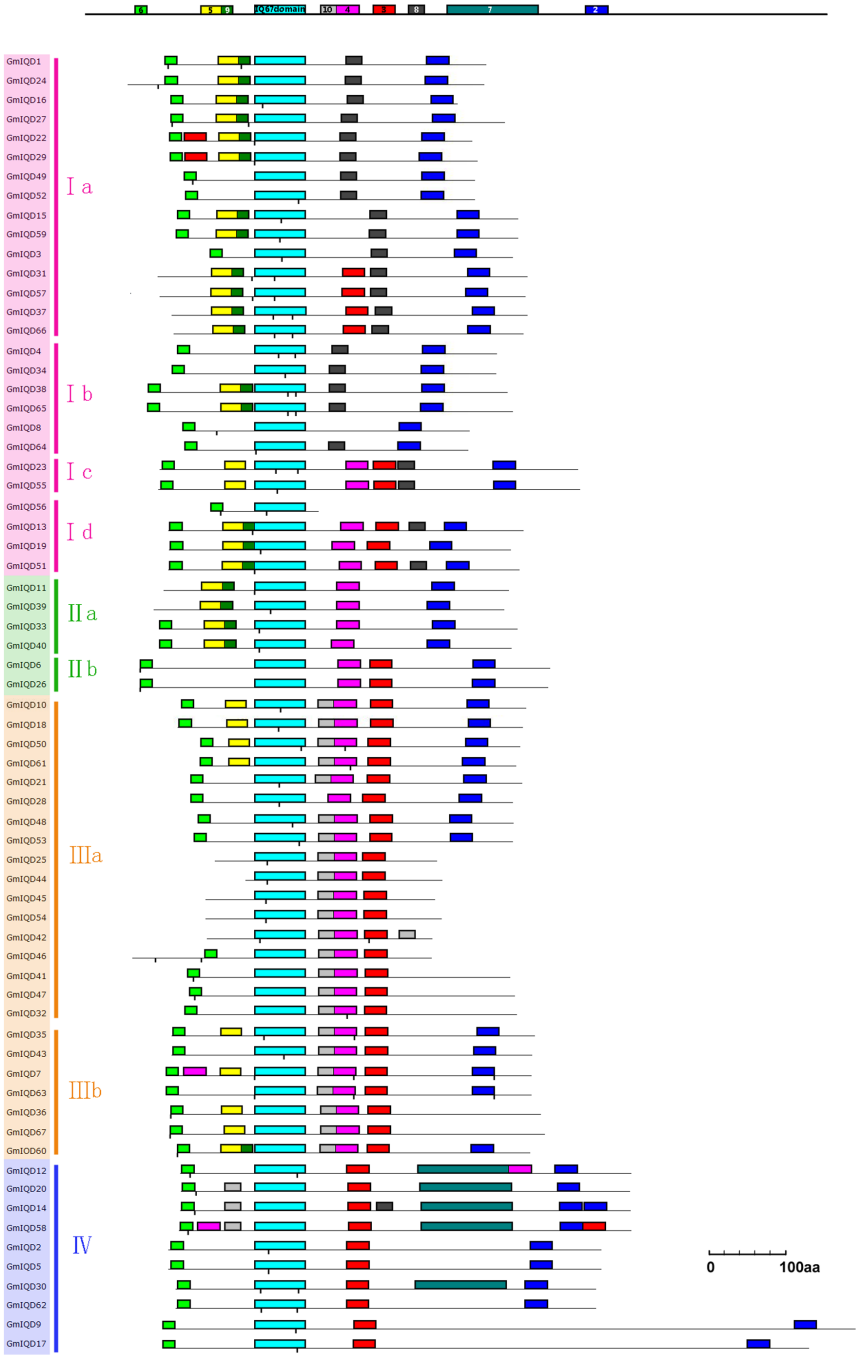
Motif patterns in 67 IQD proteins of soybean. The schematic soybean IQD proteins were aligned relative to the IQ67 domain (motif 1, light blue box). The lengths of the proteins and motifs can be estimated using the scale. Motifs shared by at least four soybean IQD proteins are depicted at the reference bar on top of each alignment. The positions of putative calmodulin-binding sites predicted by the Calmodulin Target Database are indicated by vertical tick marks below each protein model. Subfamilies and subclasses (I–IV) of IQD proteins are highlighted with colored backgrounds and colored vertical bars as in Figure 1a to the right of the gene names.

To infer the similarity and evolutionary ancestry of soybean IQD proteins, we constructed an unrooted phylogenetic tree of the 67 soybean protein sequences. The soybean IQD gene family was categorized into four major subfamilies (subfamily I, II, III and IV; Figure 1a) according to phylogenetic analysis of IQD genes in *Arabidopsis*, rice, tomato and *Brachypodium distachyon* [30], [34], [35]. Subfamily I was further divided into four subclasses (clade Ia, Ib, Ic and Id), and subfamily II and III were divided into two subclasses (clade IIa and IIb; clade IIIa and IIIb) based on bootstrap values, the existence and positions of introns and the presence of protein motifs flanking the IQ67 domain (Figure 1 and 2). Subfamily I (containing 27 members) is the largest group, followed by subfamily III (24) and subfamily IV (10). Subfamily II has the fewest IQD gene members (6). This distribution pattern is similar to that observed for IQD genes in *Arabidopsis* and rice (Table 2). The phylogenetic tree reveals that 62 of the 67 soybean IQD genes form 31 gene pairs with strong bootstrap values (Figure 1a).

To further examine the structural diversity of the IQD genes in soybean, we deduced the exon/intron organization of individual GmIQD genes (Figure 1b). A comparison of the 67 genomic loci with corresponding cDNA sequences revealed that most of the gene models predicted by GSDS are correct, except for one pair of genes (*GmIQD9*/-*17*). Both *GmIQD9* and *GmIQD17* encode six exons, but GSDS predicted that these genes contain only five exons. This unconformity is caused by the missing annotation of the fifth intron by GSDS. The schematic structures reveal that the coding sequence of each IQD gene contains 2–6 translated exons (Figure 1b), which is similar to that reported in *Arabidopsis*, rice and *Brachypodium distachyon* [30], [34]. More than three–fifths of the soybean IQD gene family (41 members) contain five or six protein-coding exons, and one gene (*GmIQD56*, encoding the smallest protein, comprising 141 aa) contains two exons (Figure 1b). Most closely related soybean IQD members in the same subfamilies share similar intron numbers and exon lengths. Soybean IQD genes in subfamily II and IV possess five and six exons, respectively. Most members in subfamily III contain five exons, except for *GmIQD44* (four exons) and *GmIQD32*, *GmIQD41*, *GmIQD46* and *GmIQD47* (six exons). Subfamily I genes harbor 2–5 exons. All introns of most IQD genes are in phase-0 (interrupting two triplet codons exactly); a phase-1 intron (separating the first and second nucleotides of a codon) was found in 15 remaining IQD genes, and no phase-2 intron (splitting the second and third nucleotides of a codon) was found (Figure 1b).

The exon/intron organization of 31 paralogous pairs that clustered together at the terminal branch of the phylogenetic tree was further examined to obtain traceable intron gain/loss information. Although twenty-seven paralogous pairs exhibited conserved exon/intron structures, four pairs (*GmIQD16*/-*27*, *GmIQD38*/-*65*, *GmIQD25*/-*44* and *GmIQD42*/-*46*) showed certain variations (Figure 1b). These differences may have been derived from single intron loss or gain events during the long evolutionary period. Based on analysis of the exon/intron organization of IQD genes from soybean, *Arabidopsis* [30], rice [30], and *Brachypodium distachyon* [34], we infered that both *GmIQD16* and *GmIQD38* gained the third intron; *GmIQD46* gained the first intron while *GmIQD44* lost the first intron. The second or third exons in the central regions of most members encode amino acids 17–67 of the IQ67 domain, except for *GmIQD46* (the fourth exon) and *GmIQD56* (the C-terminal exon), with a conserved phase-0 intron separating codons 16 and 17 (Figure 1b and Figure S1).

A total of 67 IQD genes from soybean were subjected to analysis with MEME to reveal conserved domains or motifs shared among related proteins. We identified 10 conserved motifs (Figure 2 and Table S1). Each of the putative motifs was annotated by searching Pfam and SMART. Motif 1 was found to encode the IQ domain. Motif 2 and motif 7 were found to encode proteins of unknown function (DUF4005) and (DUF3982). While the other subfamily-specific motifs have not functional annotation. As expected, most of the closely related members had common motif compositions, suggesting functional similarities among IQD proteins within the same subfamily (Figure 2). The most common motif is motif 1, found in all sixty-seven soybean IQD genes (Figure 2). Motif 8 is mainly present in subfamily I besides one of *GmIQD14* exists in subfamily IV. Subfamily III members contain motif 1, motif 10, motif 4 and motif 3 in order, except for *GmIQD28* lacking motif 10. Motif 7 is peculiar to subfamily IV. To some extent, these subfamily-specific motifs may contribute to the functional divergence of IQD genes in soybean. The detailed information is shown in table S1. To predict calmodulin-binding sites, we searched the Calmodulin Target Database, which provides various structural and biophysical parameters for the 67 soybean IQD protein sequences. This analysis predicted that all soybean IQD proteins contain multiple IQ motifs and 1–3 strings of high-scoring amino acid residues (Table 3). These IQ motifs and amino acid residues indicate the locations of putative calmodulin interaction sites. Among the 67 IQD protein sequences, the predicted calmodulin binding sites of 50 sequences overlap with the IQ67 domain (Figure 2).

**Table 3:** Predicted calmodulin binding sites in soybean IQD proteins.

Group	Name	Gene Identifier	Predicted calmodulin binding sequence	
Ia	GmIQD1	Glyma01g01030	7-WVKSLFGIRREKEKKLN	100-V**AVVRLTSQGRGRTMFG**
	GmIQD3	Glyma01g42620	94-VRGH**IERK**RTAEW	
	GmIQD15	Glyma05g03450	136-LVRG**HIERKR**TAEWL	
	GmIQD16	Glyma05g35920	120-G**QERLAVVKIQT**FFR	
	GmIQD22	Glyma07g01760	109-FSGS**REKWA**AVKI	
	GmIQD24	Glyma07g14910	39-MGRATRW**VKSLFGIRKE**	
	GmIQD27	Glyma08g03710	2-GRAIRWLKGLFGIRTDRER	102-RDTTFGGAGQERL**AVVKI**
			164-LIRAQATVRSKKSRNEAHR	
	GmIQD29	Glyma08g21430	108-FSGS**REKWA**AVKI	
	GmIQD31	Glyma09g26630	123-RRVAEETTA**AAVKIQSAFR**	153-K**ALVKLQALVRGHIVRKQT**
	GmIQD37	Glyma10g38310	136-ALVKLQALVRGHIVRKQS	158-**RRMQTLVRLQAQARASRA**
	GmIQD49	Glyma13g43031	8-**LKG LLGKKKEKDYCGY**	
	GmIQD52	Glyma15g02370	148-**AQAVARSVRARRSM**	
	GmIQD57	Glyma16g32161	121-RVANETTA**AAVKIQSAFRG**	150-K **ALVKLQALVRGHIVRKQT**
	GmIQD59	Glyma17g14000	137-LVRG**HIERKR**TAEW L	
	GmIQD66	Glyma20g29550	133-LKALVKLQALVRGHIVRKQS	155-**RRMQTLVRLQAQARASRA**
Ib	GmIQD4	Glyma02g00710	133-LQALVRGHLVRKQ**A**RETL	155-AL**VIAQ**SRARAQRA
	GmIQD8	Glyma03g40630	46-RR**WSFGKLTGAGH**KF	
	GmIQD34	Glyma10g00630	148-LVRK**QARETLRCIQ**ALVIA	
	GmIQD38	Glyma10g39030	181-RKQ**AKATL**RC	193-ALVTAQ
	GmIQD64	Glyma19g43300	92-KDK**NKAATKIQA**SF	
	GmIQD65	Glyma20g28800	182-RKQ**AKATL**RC	194-AL**VTAQ**AR
Ic	GmIQD23	Glyma07g05680	152-LV**KLQALVRGHNVR**KQA	180-RVQARVLDQRIRSSL
	GmIQD55	Glyma16g02240	154-LV**KLQALVRGHN**VR	
Id	GmIQD13	Glyma04g41380	109-YGRQ**SKEERAAILIQ**SYYR	
	GmIQD19	Glyma06g13470	119-IL**IQSYYRGYL**ARRALRALKG	
	GmIQD51	Glyma14g25860	111-RQ**SKEERAATLIQ**SYYRGYLARRALRAL	
	GmIQD56	Glyma16g22935	13-RGRFLRSS	73-GHLARR**AYKALKSLVKLQA**LVR
IIa	GmIQD11	Glyma04g23760	119-K**IQESSAIKIQIAFRGY**L	
	GmIQD33	Glyma09g35920	125-IKESA**AAIKIQTAFRG**Y	
	GmIQD39	Glyma11g20880	132-KIQES**SAIKIQTAYRGYL**A	
	GmIQD40	Glyma12g01410	125-IKESA**AAIKIQTAFRG**Y	
IIb	GmIQD6	Glyma02g15590	1-**MGKKGSWFSAI**	
	GmIQD26	Glyma07g32860	1-**MGKKGSWFSAI**	
IIIa	GmIQD10	Glyma04g05520	131-VRG**RQVRKQAAVTLRCMQ**ALVRVQA	
	GmIQD18	Glyma06g05530	136-VRG**RQVRKQAAVTLRCMQ**ALVRVQAR	
	GmIQD21	Glyma07g01040	117-AIFR**GWQVRKQAAVTLR**CMQ	
	GmIQD25	Glyma07g32531	67-AYK**ARKYL**HRLR	
	GmIQD28	Glyma08g20430	117-AIFR**GWQVRKQAAVTLR**CMQ	
	GmIQD32	Glyma09g30780	205-RQEAAA**KRGRAMAY**AL	
	GmIQD41	Glyma12g31610	3-V**SGKWIKALVGLKKSEKP**G	90-R EELAAIRIQTAFRGFLA
			207-AKRERAMAYALSHQWQAG	
	GmIQD42	Glyma12g35711	68-AATRIQNAFRSFMARRTL	210-**LGKESWGWSWTERWVAAR**
	GmIQD44	Glyma13g24070	27-AYK**ARKYLH**RLRG	
	GmIQD45	Glyma13g30590	78-RAY**KARKAL**RRMKGFTK**LKI**LTEG	
	GmIQD46	Glyma13g34700	25-EIKHLIQRGWVV	90-LKR**NKRMGAK**KWF
	GmIQD47	Glyma13g38800	3-**VSGKWIKALVGLKKSEKP**	204-**AKRERAMAYALSHQWQAG**
	GmIQD48	Glyma13g42440	123-LRCMQA**LVRVQARVRA**R	
	GmIQD50	Glyma14g11050	126-VRVQARVRAR	187-GAF**K RERAIAYS**LA
	GmIQD53	Glyma15g02940	138-LRCMQA**LVRVQARVRA**R	
	GmIQD54	Glyma15g08660	78-RAY**KARKAL**RRMKGFTK**LKI**LTEG	
	GmIQD61	Glyma17g34520	197-EGA**F KR**ERAIAYSL	
IIIb	GmIQD7	Glyma03g33560	116-P**KDEVAAIKIQTAFRGY**L	227-LSKYEATTRRERALAYA
			427-NG**KAEKGSFGSAKKRL**SF	
	GmIQD35	Glyma10g05720	111-EEM**AAIRIQKAFRGYLA**	218-KLLSKYEASMRRERAMAYS
	GmIQD36	Glyma10g35721	**1-MGRKGGWFSAV**	292-**HASAKSVASQTMSV**
	GmIQD43	Glyma13g20070	126-LARR**ELR**ALRGLV	
	GmIQD60	Glyma17g23770	1-**MGKKGSWFSAV**	
	GmIQD63	Glyma19g36270	116-P**KDEVAAIKIQTAFRGY**L	227-LSKYEATMRRERALAYA
			427-NA**KAEKGSFGSAKKRL**SF	
	GmIQD67	Glyma20g31810	1-**MGRKGGWFSAV**	293-**HASAKSVASQTMSV**
IV	GmIQD2	Glyma01g05100	130-LARQ**TFK**KLEGV	175-RGYNVRRS
	GmIQD5	Glyma02g02370	130-LARR**TLQ**KLKGV	
	GmIQD9	Glyma04g02830	173-QA**IIKMQILVRARR**AR	
	GmIQD12	Glyma04g34150	13-LFGKKSS**K SNI**SK	153-KLQALVRGGRIRQS
	GmIQD14	Glyma05g01240	19-SKS**NISKGRE**KLV	
	GmIQD17	Glyma06g02841	175-II**KMQILVRARRA**WQ	
	GmIQD20	Glyma06g20341	20-KS**NIS**KGRE	
	GmIQD30	Glyma08g40880	113-QAA**IRGYQ**ARG	163-LARGYKVRHS
	GmIQD58	Glyma17g10660	12-VL**FGKKSSKSNI**SK	
	GmIQD62	Glyma18g16130	115-IRGYQARGTFKTL	161-QA**LARGYKVRHS**DV

Predicted calmodulin binding sites obtained from the Calmodulin Target Database are shown for strings of amino acid residues with a score of at least 7. Residues with the highest score (9) are highlighted in bold. Numbers before strings indicate the location of the first amino acid residues of the strings in soybean IQD protein sequences.

### Chromosomal location and gene duplication

**Figure 3:**
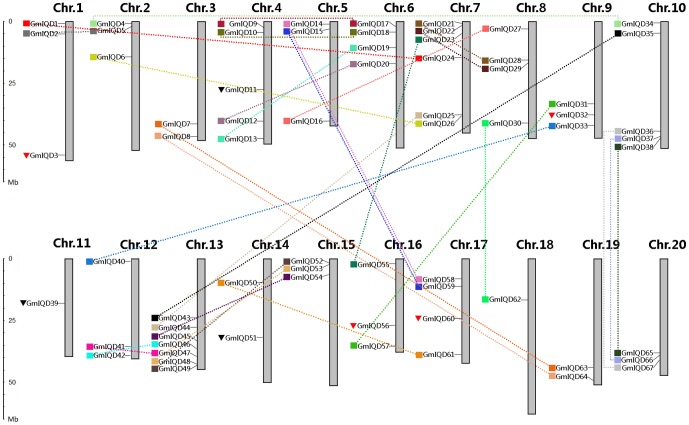
Chromosomal distribution and segmental duplication events for soybean IQD genes. The 67 IQD genes were mapped to the 20 soybean chromosomes. The duplicated paralogous pairs of IQD genes in the segmental duplicated blocks are indicated with small boxes of the same color and connected by dashed lines of the same color. Red triangles represent soybean IQD genes located on duplicated segments with the corresponding members lost. Black circles indicate soybean IQD genes not located in duplicated regions. Scale represents the length of the chromosome.

The 67 soybean IQD genes were mapped to all 20 soybean chromosomes. The distribution of soybean IQD genes varies depending on the chromosome and appears to be unequal. Both chromosomes 11 and 18 contain only one soybean IQD gene, while chromosomes 13, which possesses seven IQD genes, has the highest number of IQD genes per chromosome. Although high densities of IQD genes were found on some chromosomal regions, for instance, the bottom of chromosome 13, these is no substantial clustering of soybean IQD genes on the map (Figure 3).

We investigated gene duplication events to further understand the expansion mechanism of the soybean IQD family. Except for three genes (*GmIQD11*, *-39* and *-51*) located outside of a duplicated block, 64 genes were mapped onto 48 related duplicated blocks (Figure 3 and Table S2). Among these, twenty-two block pairs retained thirty GmIQD gene pairs, whereas the remaining four duplication blocks harbor *GmIQD3*, -*32*, -*56* and -*60* respectively, but lack IQD sisters in their corresponding synteny blocks (Figure 3 and Table S2). Analysis of GmIQD paralogous pairs showed that one pair (*GmIQD11*/-*39*) appear to be closely related paralogs, sharing 91.2% identity (Table S3) as well as similar exon–intron organization. However, both of them exist outside of any duplicated blocks. Except for *GmIQD11*/-*39*, 30 out of 31 gene pairs have remained in conserved positions on segmental duplicated blocks, indicating that these genes were generated by segmental duplication. Furthermore, we analyzed the adjacent genes to determined whether tandem duplication has taken place. A pair of genes separated by three or fewer genes within a 100-kb region on a chromosome may have resulted from tandem duplication. According to this criterion, no pair was found to be generated by tandem duplication. Therefore, segmental duplication appears to have played a crucial role in the expansion of the IQD gene family in soybean (Figure 3 and Table S2).

To explore the selective constraints on duplicated soybean IQD genes, we calculated the ratio of nonsynonymous versus synonymous substitutions (Ka/Ks) for each pair of duplicated IQD genes. In general, a ratio of 1 indicates that both genes are drifting neutrally; a Ka/Ks ratio >1 indicates accelerated evolution with positive selection, while a ratio <1 indicates functional constraint, with negative or purifying selection of the genes[44]. The Ka/Ks ratios from 31 soybean IQD paralogous pairs (Table 4) were less than 0.6. This result suggests that the soybean IQD gene family has evolved mainly under the influence of strong purifying selection pressure, with limited functional divergence occurring after segmental duplication. Duplication of these 31 paralogous pairs was estimated to have occurred between 6.39 to 17.94 Mya (Table 4), according to the divergence rate of 6.1×10^−9^ synonymous mutations per synonymous site per year, as previously proposed for soybean [45], [46].

**Table 4:** Divergence between paralogous IQD gene pairs in soybean.

Group	No.	Paralogous pairs	Ka	Ks	Ka/Ks	Duplication date (MY)	Duplicate type
Ia	1	GmIQD1-GmIQD24	0.0474	0.0802	0.5914	6.57	S
	2	GmIQD16-GmIQD27	0.045	0.195	0.228	15.99	S
	3	GmIQD22-GmIQD29	0.029	0.108	0.267	8.83	S
	4	GmIQD49-GmIQD52	0.041	0.106	0.388	8.66	S
	5	GmIQD15-GmIQD59	0.041	0.158	0.258	12.96	S
	6	GmIQD31-GmIQD57	0.029	0.147	0.194	12.03	S
	7	GmIQD37-GmIQD66	0.030	0.116	0.260	9.48	S
Ib	8	GmIQD4-GmIQD34	0.044	0.124	0.356	10.18	S
	9	GmIQD38-GmIQD65	0.054	0.111	0.485	9.07	S
	10	GmIQD8-GmIQD64	0.039	0.134	0.293	11.00	S
Ic	11	GmIQD23-GmIQD55	0.017	0.086	0.193	7.08	S
Id	12	GmIQD13-GmIQD19	0.057	0.164	0.346	13.47	S
IIa	13	GmIQD11-GmIQD39	0.043	0.093	0.460	7.60	O
	14	GmIQD33-GmIQD40	0.022	0.094	0.238	7.70	S
IIb	15	GmIQD6-GmIQD26	0.022	0.091	0.245	7.49	S
IIIa	16	GmIQD10-GmIQD18	0.030	0.152	0.197	12.43	S
	17	GmIQD50-GmIQD61	0.031	0.162	0.189	13.30	S
	18	GmIQD21-GmIQD28	0.041	0.125	0.325	10.22	S
	19	GmIQD48-GmIQD53	0.029	0.111	0.262	9.08	S
	20	GmIQD25-GmIQD44	0.052	0.157	0.335	12.84	S
	21	GmIQD45-GmIQD54	0.034	0.105	0.325	8.57	S
	22	GmIQD42-GmIQD46	0.058	0.219	0.263	17.94	S
	23	GmIQD41-GmIQD47	0.037	0.095	0.387	7.78	S
IIIb	24	GmIQD35-GmIQD43	0.033	0.114	0.293	9.34	S
	25	GmIQD7-GmIQD63	0.024	0.093	0.253	7.61	S
	26	GmIQD36-GmIQD67	0.045	0.127	0.349	10.43	S
IV	27	GmIQD12-GmIQD20	0.054	0.134	0.400	10.99	S
	28	GmIQD14-GmIQD58	0.035	0.118	0.297	9.66	S
	29	GmIQD2-GmIQD5	0.059	0.109	0.537	8.93	S
	30	GmIQD30-GmIQD62	0.067	0.151	0.443	12.35	S
	31	GmIQD9-GmIQD17	0.028	0.078	0.363	6.39	S

S: segmental duplication, O: other events.

### Comparative analysis of the IQD genes in soybean, *Arabidopsis*, rice, tomato and *Brachypodium distachyon*

**Figure 4:**
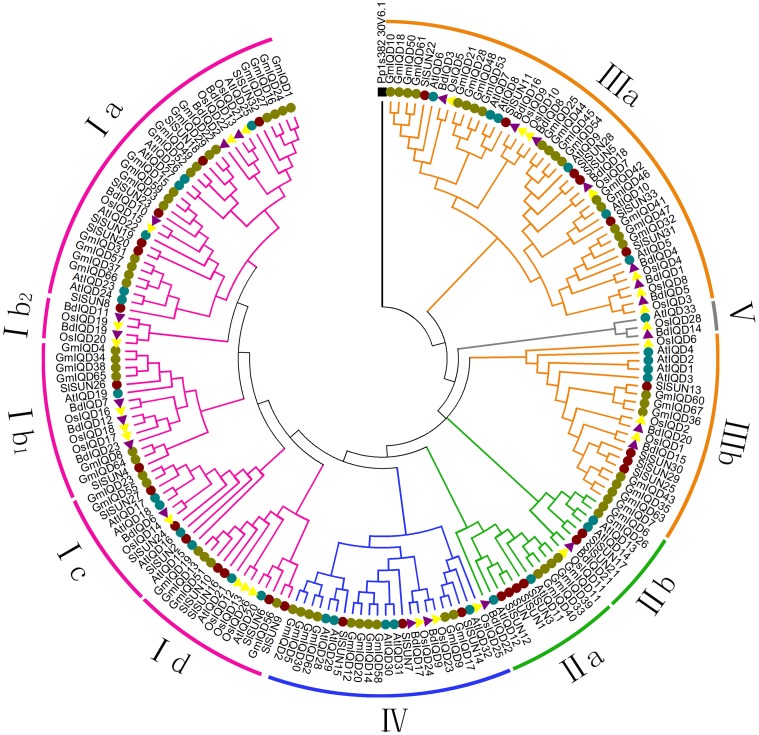
Phylogenetic tree of full-length IQD proteins from soybean, *Arabidopsis*, rice, tomato and *Brachypodium distachyon*. The tree was generated with Clustal X2.0 using the NJ method. Dicotyledons (soybean, tomato and *Arabidopsis*) IQD proteins are marked with colored dots. Monocotyledons (rice and *Brachypodium distachyon*) are marked with colored triangles. A moss IQD protein (Pp1s38230v6), used as the outgroup, is marked with a black box. Each IQD subfamily is indicated by a specific color.

The development of comparative genomics has enabled the analysis of the same protein families among different species. We constructed an NJ phylogenetic tree using 184 full-length protein sequence to reveal the evolutionary relationships among soybean, *Arabidopsis*, rice, tomato and *Brachypodium distachyon* IQD proteins [34]. In *Arabidopsis*, the IQD gene family is divided into four subfamilies, with *AtIQD33* (containing a C-terminally truncated IQ67 domain) as the outgroup. Therefore, based on their phylogenetic relationships, the combined phylogenetic tree can be divided into five distinct subfamilies (I to V; Figure 4) [30]. In general, IQD I genes comprise the largest subfamily in these plant species, except for *Brachypodium distachyon*, where both IQD I and III comprise the largest subfamilies. By contrast, IQD V genes comprise the smallest IQD subfamily (Figure 4, Table 2).

To illustrate the paralogous and orthologous relationships among IQD family members, the subfamilies were further divided into subgroups using previously defined clades from studies of *Arabidopsis*, rice and tomato IQDs, as shown in Figure 4. IQD subfamily I was divided into four subclasses, i.e., a, b, c and d, and clade b was further divided into two clades, b_1_ and b_2_. Because one of the IQD Ib clades only contains four IQD genes (*BdIQD11*, *BdIQD19*, *OsIQD19* and *OsIQD20*) from monocots, we assigned these four genes to the rice- and *Brachypodium distachyon-*specific Ib_2_ clade. The clade containing the genes encoding C-terminal IQ67 domains was defined as Id. Notably, no members of *Brachypodium distachyon* were detected in this clade, suggesting that *Brachypodium distachyon* IQD family lost its members of this subgroup during the long period of evolution. Both IQD II and IQD III subfamilies were divided into two subclasses, a and b, which were designated as described by Zejun et al.(2013) and Abel et al. (2005) [35]. The C-terminally truncated IQ67 domain-containing genes (*At IQD33*, *OsIQD28* and *BdIQD14*) comprise IQD V subfamily (Figure 4) [30], [34].

The combined phylogenetic tree reveals that most genes in the IQD family, especially the duplicated genes, are contained in paralogous pairs in each species, which supports the occurrence of species-specific IQD gene duplication events. By contrast, we identified 20 pairs of orthologous genes from monocotyledons (rice and *Brachypodium distachyon*) distributed among all of the subfamilies. In addition, two pairs of orthologous genes from dicotyledons (soybean and tomato) stemming from subfamily I (*GmIQD56* and *SlSUN9*) and subfamily III (*GmIQD60* and *SlSUN13*) were found. And *AtIQD20* and *OsIQD26*, members of subfamily I, formed a pair of orthologous genes.

### Conserved microsynteny of IQD III genes from soybean, *Arabidopsis* and tomato

The analysis of microsynteny provides valuable information for identifying gene expansion patterns and inferring gene orthology or paralogy. We combined genetic and phylogenetic analyses to perform microsynteny analysis of three dicotyledons, i.e., soybean, tomato and *Arabidopsis*.

To provide a basic framework for the identification of IQD III orthologous or paralogous genes, 44 IQD III genes, including 24 predicted soybean IQDs, 10 *Arabidopsis* IQDs and 10 tomato IQDs, were classified into four distinct clades, clade 1 (thirteen genes), clade 2 (five genes), clade 3 (eleven genes) and clade 4 (fifteen genes), based on phylogenetic analysis (Figure S2). Clade 1, 2 and 3 correspond to IQD IIIa and clade 4 corresponds to IQD IIIb (Figure 4 and S2). Each clade contains at least one gene from soybean, tomato and *Arabidopsis*, indicating that members from different species may be derived from a common ancestor.

**Figure 5:**
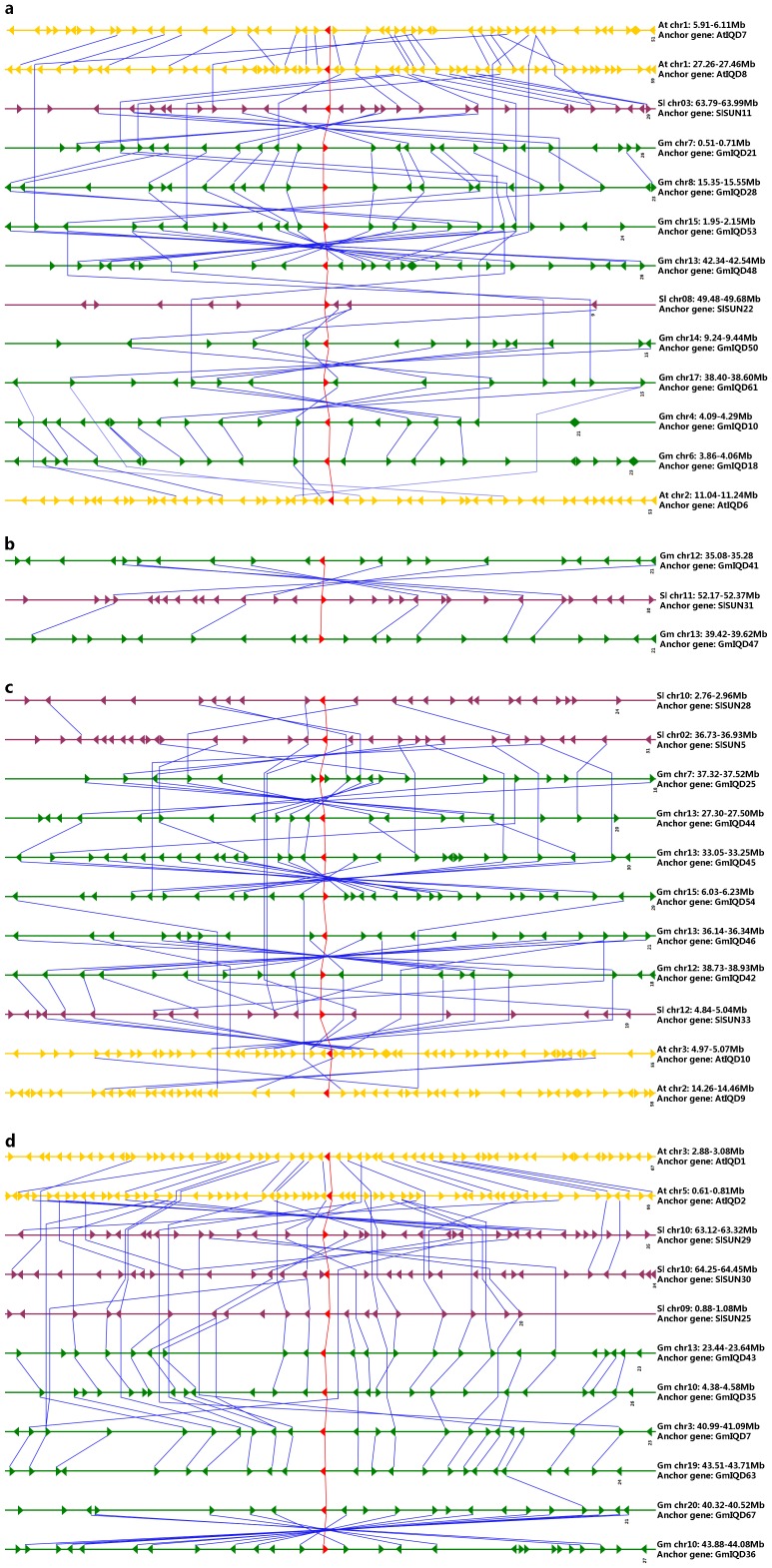
Microsynteny map of IQD III genes in soybean, *Arabidopsis* and tomato. **a, b, c** and **d:** four groups of syntenic segments. The relative positions of all flanking protein-coding genes were defined by the anchored IQD III genes (red). Colored horizontal lines represent chromosome segments of soybean (green), *Arabidopsis* (yellow) and tomato (purple). Triangles of the same color represent individual genes and their transcriptional orientations. The total number of genes on each segment is indicated to the right below the segment. Colored lines connect the conserved gene pairs among the segments (anchor gene pairs, red; others, blue).

Subsequently, to produce a comparative genetic map, 44 IQD III genes from the three dicot genomes were used as anchor genes. Conserved microsynteny was identified through reciprocal pairwise comparisons of the chromosomal regions containing IQD III genes. Microsynteny relationships among *AtIQD3*, *AtIQD4, AtIQD5*, *GmIQD32*, *GmIQD56* or *SlSUN13* with other IQD III members in these three dicot genomes were not observed. The map reveals that the 38 conserved syntenic segments diverged into four groups (Figure 5), which were anastomosed with the classification revealed by phylogenetic tree analysis.

In clade 1 (Figure 5a), Map a shows a higher level of microsynteny, with both the same and opposite directions. *SlSUN11*/*GmIQD21* and *GmIQD53*/*GmIQD48* exhibit remarkable opposite-direction microsynteny, while *GmIQD10*/*GmIQD18, GmIQD21*/*GmIQD28*, *AtIQD7*/*AtIQD8* and *AtIQD8*/*SlSUN11* are aligned with flanking gene pairs in the same order but discordant transcriptional orientation. In addition, genes in map a were divided into two groups (Figure 5a), i.e., one group with higher levels of microsynteny (*GmIQD21*, *GmIQD28*, *GmIQD53*, *GmIQD48*, *AtIQD7*, *AtIQD8* and *AtIQD8* and *SlSUN11*) and the other group with lower levels of microsynteny (*GmIQD61*, *GmIQD50*, *GmIQD10*, *GmIQD18*, *AtIQD6* and *SlSUN22*). These two groups were also detected in the phylogenetic tree of IQD III genes (Figure S2). In clade 2 (Figure 5b), two pairs from soybean and tomato, *GmIQD41*/*SlSUN31* and *GmIQD47*/*SlSUN31*, exhibited microsynteny. However, the predicted duplicated pair *GmIQD41*/*GmIQD47* had no detectable linkage with each other. High level of microsynteny exists in Clade 3, with most pairs in reverse order (Figure 5c), especially *GmIQD42*/*GmIQD46*, *GmIQD45*/*GmIQD54* and *GmIQD25*/*GmIQD44*. *GmIQD45*/*GmIQD44* and *GmIQD44*/*SlSUN33* were identified as having same-direction microsynteny. In clade 4 (Figure 5d), we also observed a higher level of microsynteny. Except for *AtIQD2/SlSUN29*, *SlSUN30*/*SlSUN29* and *GmIQD67*/*GmIQD36*, which are aligned in the opposite direction, most gene pairs in this clade have successive collinearity in order and the same orientation.

Two regions are considered to have originated from a large-scale duplication event when five or more protein-coding gene pairs flanking the anchor point are ligatured with the best non-self match (E-value <1e−10) [47], [48]. Applying this standard, except for the pair *GmIQD41*/*GmIQD47*, all soybean IQD III paralogous gene pairs were generated from a large-scale duplication event, which further supports the results of soybean gene duplication analysis (Figure 5, Table S4).

To estimate the degree of conserved gene content and order, the synteny quality was calculated [47]. The average synteny quality of IQD III genes from the three dicotyledons genomes was 18.41% (Table S5d). Due to the large number of syntenic genes between tomato and soybean, the synteny quality between these genomes is 26.39%; this value is higher than that observed in the Sl/At synteny blocks (16.68%). The lowest synteny quality (12.15%) was found between soybean and *Arabidopsis* (Table S5). Details of this comparative analysis are shown in Table S5.

### Expression patterns of soybean IQD genes in various tissues

**Figure 6:**
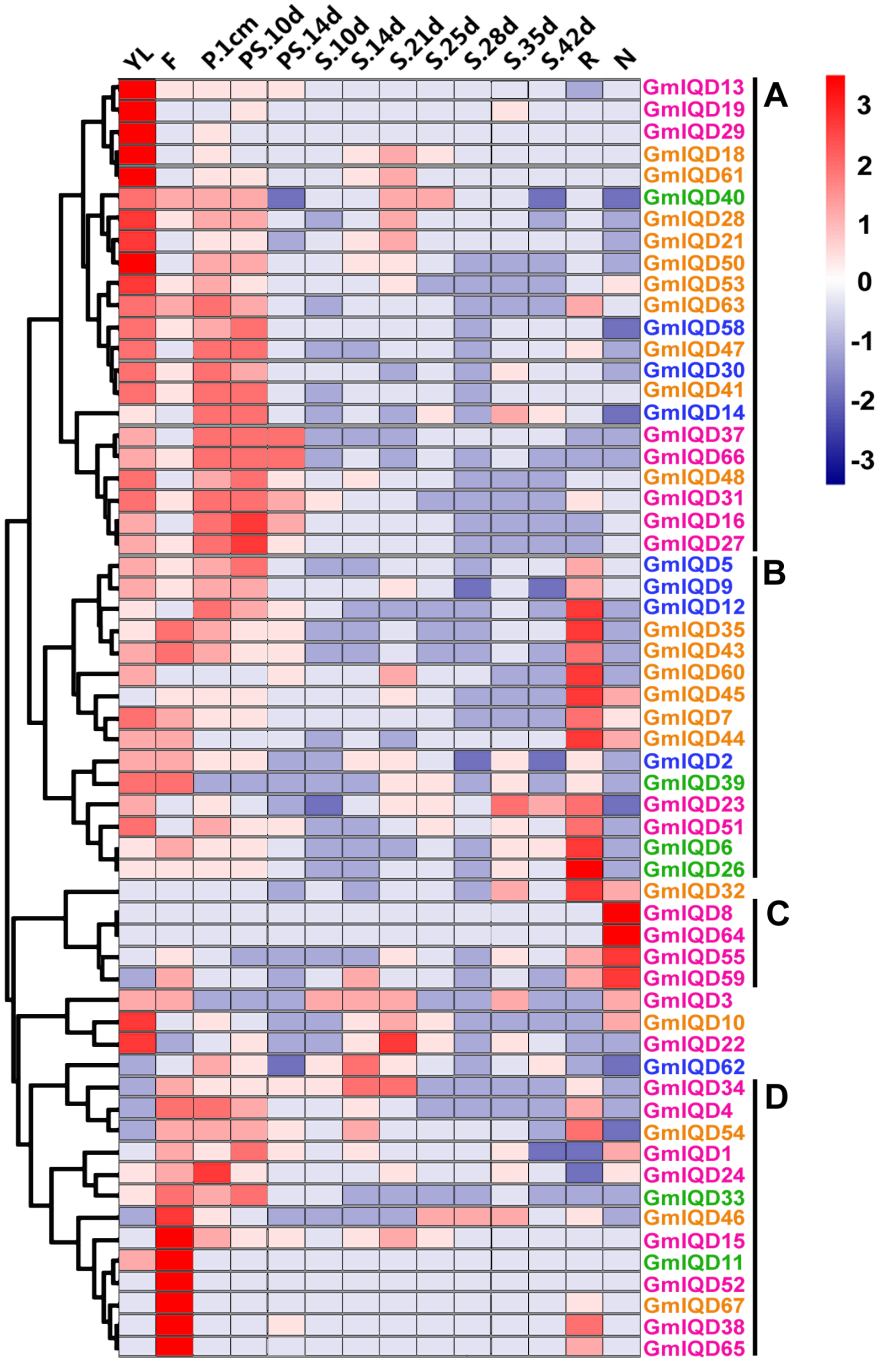
Hierarchical clustering of the expression profiles of soybean IQD genes in 14 tissues. RNA-seq relative expression data from 14 tissues were used to reconstruct the expression patterns of soybean genes. The raw data was normalized and retrieved from the online database http://soybase.org/soyseq/. The normal relative expression levels of 67 IQD genes are shown in Table S6. YL, young leaf; F, flower; P.1cm, one cm pod; PS.10d, pod shell 10 DAF; PS.14d, pod shell 14 DAF; S.10d, seed 10 DAF; S.14d, seed 14 DAF; S.21d, seed 21 DAF; S.25d, seed 25 DAF; S.28d, seed 28 DAF; S.35d, seed 35 DAF; S.42d, seed 42 DAF; R, root; N, nodule. Gene names in different subfamilies are highlighted with various colors. Genes clustered into four groups (A–D) are indicated by the black vertical bars.

To gain insight into the expression patterns of soybean IQD genes in various tissues, we searched the RNA-Seq Atlas of *Glycine max*; this atlas provides high-resolution gene expression data from 14 diverse tissues, including aerial tissues (young leaf, flower, one-cm pod, pod-shell 10-DAF and pod-shell 14-DAF), underground tissues (root and nodule) and seed tissue at various stages of development (seed 10-DAF, seed 14-DAF, seed 21-DAF, seed 25-DAF, seed 28-DAF, seed 35-DAF and seed 42-DAF). Because the expression profiles of eight IQD genes (*GmIQD17*, *-20*, *-25*, *-36*, *-42*, *-49*, *-56*, *-57*) weren't obtained in the database, we only examined the expression patterns of fifty-nine IQD genes (Figure 6 and Table S6).

Most soybean IQD genes exhibit broad expression patterns (Figure 6). Forty-four soybean IQDs are expressed in all of the seven tissues (young leaves, flowers, one-cm pod, pod-shell, roots, nodules and seed). The heat map also revealed that the majority of GmIQDs showed preferential expression. Based on a hierarchical clustering analysis, fifty-nine IQD genes were mainly clustered into four groups (A–D) (Figure 6). Group A showed partial expression in young leaves, group B in roots, group C in nodules and group D in flowers. Eight GmIQDs (*GmIQD8*, -*13*, -*19*, -*29*, -*36*, -*52*, -*64* and -*67*) showed marked high transcript abundance profiles in only a single tissue. Among the fifty-nine soybean IQD genes examined, six showed the highest transcript accumulation in young leaves (*GmIQD13*, -*18*, -*19*, -*29*, -*50* and *-61*), six in flowers (*GmIQD11*, -*15*, -*38*, -*52* -6*5* and -*67*), one in roots (*GmIQD26*) and two in nodules (*GmIQD8* and -*64*; Figure 6). Genes in different subfamilies have their primary abundant transcripts, for instance, GmIQD I in leaves, flowers and nodules, GmIQD II in flowers and roots, GmIQD III in young leaves, flowers and roots and GmIQD IV in roots and young leaves (Figure 6). These subfamily-specific tissue expression patterns may be closely related to gene functions. The expression patterns of the paralogous pairs were also revealed by heat maps; paralogous pairs with high sequence similarity have similar expression patterns. The best examples of this include *GmIQD8*/-*64* and *GmIQD6*/-*26*, which are strongly expressed in nodules and root respectively, with little or no expression in other tissues. Expression divergence was also found in paralogous pairs. For example, *GmIQD15* is highly expressed in flowers, while its paralog, *GmIQD59*, is highly expressed in nodules.

### Examination of soybean IQD gene expression by qRT-PCR

Since soybean production is limited by stress, it is important to identify the master regulators of stress responses in soybean, as well as their regulatory pathways. According to microsynteny analysis, the high level of microsynteny indicates that IQD III genes existed before the divergence of the three dicotyledon genomes examined (soybean, tomato and *Arabidopsis*). In addition, IQD III genes in the same clade may share common ancestors and play similar roles in these species. *AtIQD1*, which belongs to the IQD III subfamily, plays a major role in the response to biotic stress, as it mediates the accumulation of glucosinolate in response to phytophagous insect attack. Jasmonic acid methyl ester (MeJA), the plant hormones and the signal molecules, widely exists in plants, which triggers expression of plants defense genes by exogenous applications and has similar effects with mechanical damage and insect herbivory [49], [50]. Based on these, we subjected 24 members of the soybean IQD III subfamily to real-time quantitative PCR (qRT-PCR) analysis to examine their regulation by MeJA.

**Figure 7:**
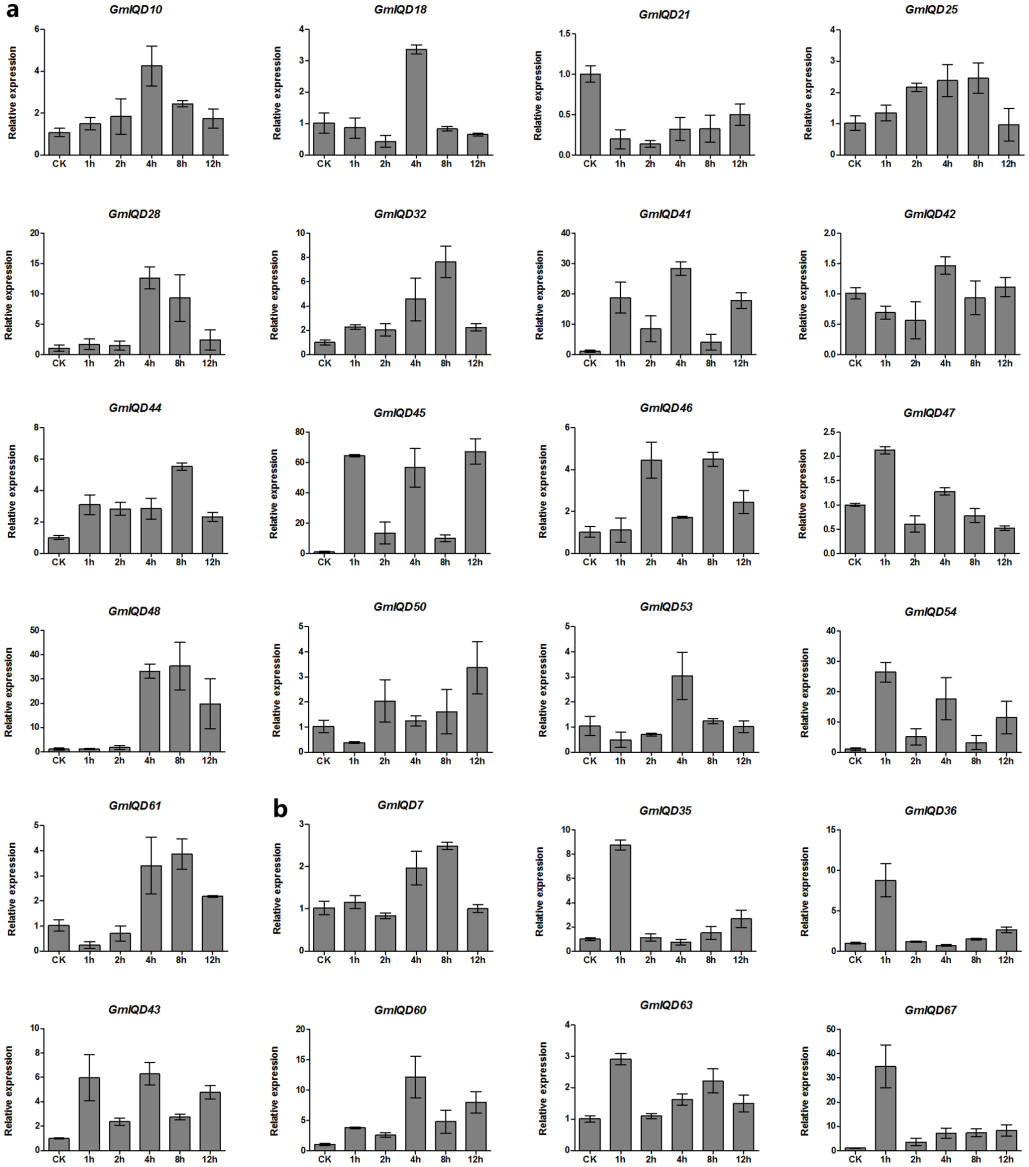
Expression patterns of 24 selected IQD III genes under MeJA stress using qRT-PCR. Relative expression levels of 24 IQD genes were examined by qRT-PCR and normalized to the expression of *CYP2*. Bars represent standard deviations (SD) of three biological replicates. Y-axes indicate the scale of the relative expression levels. X-axes show time courses of MeJA stress treatments for each gene. **a** and **b**: data for genes from IIIa and IIIb, respectively.

The qRT-PCR results show that all 24 genes are MeJA-responsive, but some differences were observed among these genes (Figure 7). Although 23 genes were upregulated by MeJA treatment, *GmIQD21* was obviously downregulated (<0.5 folds) at all time points. Eleven of the twenty-three upregulated GmIQD III genes exhibited minor changes in expression (relative expression scale from 0 to 5 and lower), including *GmIQD10*, -*18*, -*21*, -*25*, -*42*, -*47*, -*53*, -*61*, -*7*, -*36* and -*63*. By contrast, 12 genes (*GmIQD2*, -*32*, -*41*, -*44*, -*45*, -*46*, -*48*, -*50*, -*53*, -*54*, -*35*, -*43*, -*60* and -*67*) exhibited major changes in expression (relative expression scales from 0 to 5 up 0 to 80). The expression of six genes (*GmIQD35*, -*36*, -*47*, -*54*, -*63* and -*67*) peaked relatively early (at 1 h of treatment); *GmIQD54* and -*67* were strongly upregulated (more than 26-fold and 34-fold, respectively). Eight genes (*GmIQD10*, -*18*, -*28*, -*41*, -*42*, -*43*, -*53* and *-60*) were highly expressed at 4 h; *GmIQD28* and *-60* had the highest expression level more than 12-fold and *GmIQD41* had the highest expression level more than 28-fold. While seven genes (*GmIQD7*, -*25*, -*32*, -*44*, -*46*, -48 and *-61*) exhibited the highest expression levels at 8 h; *GmIQD48* were strongly induced by more than 35-fold. Only one gene (*GmIQD45*) had the highest expression level at 12 h, with a relative expression level approaching 70-fold. We also compared the expression profiles of paralogous pairs. Most paralogs in a pair had different expression profiles. For example, the expression of *GmIQD28* peaked at 4 h while its sister gene, *GmIQD21*, was downregulated at all time points, suggesting that these genes may play diverse roles in the response to MeJA stress.

## Discussion

### Structural characteristics of IQD proteins

The plant-specific IQD gene family has previously been comprehensively analyzed in *Arabidopsis*, rice, tomato and *Brachypodium distachyon*, this gene family has not been previously identified and annotated in soybean. We identified and characterized 67 IQ67 domain-encoding genes in soybean using genome-wide analysis. The IQD gene family in soybean is by far the largest one compared to that in other plant species (33 in *Arabidopsi*s, 29 in rice, 34 in tomato and 23 in *Brachypodium distachyon*). At ∼1,150 Mbp, with ∼46,400 predicted coding genes, soybean possesses 9.2-fold larger genome size and 1.75-fold higher gene count than *Arabidopsis*, which has a genome of 125 Mbp and ∼26,500 coding genes [51]. Given the obvious difference in genome size and estimated gene count between soybean and *Arabidopsis*, the IQD genes in soybean seems to be highly expanded. The presence of twice as many of these genes in soybean versus *Arabidopsis* may be mainly due to the recent polyploidy event and segmental duplication events in soybean's evolutionary history. It can be speculated that the presence of more IQD genes in soybean genome may reflect the great needs for these genes coding for calcium signal regulatory components with functions in plant development, defense response or others.

The common feature of IQ67 domain proteins is the arrangement of three IQ motifs separated by 11 and 15 intervening amino acid residues (Figure S1). To date, at least five protein families containing IQ motifs, which play a role in the calcium signaling pathway, have been identified in *Arabidopsis*. These protein families include the cyclic nucleotide gated channels family (CNGC), the IQ-Motif family (IQM), the CaM-binding transcriptional activator family (CAMTA), the myosin family and the IQD family, which contain one, one, two, five and up to three IQ motifs, respectively [52]–[54]. The unique spacing of IQ motifs and exon/intron organization of each family suggest that these IQD protein families represent separate classes of putative calmodulin targets. The calmodulin-interacting peptides in AtIQD20 and CNGC proteins, which were experimentally verified, were previously predicted using the algorithm provided by the Calmodulin Target Database successfully [30]. In the current study, using the Calmodulin Target Database, we detected calmodulin-binding sites in all soybean IQD proteins, which strongly suggests that all IQD proteins have the potential to interact with calmodulin (Figure 2 and Table 3). Three aspects of IQD proteins appear to underlie the mechanism of interaction between IQD proteins and calmodulin: the number and specific composition of the IQ, 1-5-10 and 1-8-14 motifs, the predicted calmodulin binding site and the overall tertiary structure of the IQD protein.

Of the 31 soybean IQD paralogs examined, 27 exhibit highly conserved exon-intron structures, which is consistent with the high degree of position and phase conservation broadly found across angiosperms [41]. In addition, the sizes of related introns between paralogs are also highly conserved, indicating that few insertions and deletions have accumulated within introns over the past 13 million years [41]. Most introns in GmIQD genes are in phase-0. This strong bias for phase-0 introns in soybean IQD genes is also found in IQD genes of *Arabidopsis*, rice and *Brachypodium distachyon*. The strong bias for one intron phase class, along with the variation in the number of exons (two to six) and the sizes of encoded proteins, suggests that exon shuffling has played a prominent role during the evolution and diversification of IQD genes [30].

65 of 67 soybean IQD proteins have relatively high isoelectric points with an average of 10.1. It is very similar to *Arabidopsis* (10.3), rice (10.4) and *Brachypodium distachyon* (10.3) [30], [34]. The extensive presence of the basic isoelectric point and high frequency of serine residues (*Arabidopsis*: ∼11%, rice: ∼11%, *Brachypodium distachyon*: ∼11.5% and soybean: ∼12%; Table S7) in IQDs suggest that the basic nature of IQDs is crucial to their biochemical functions [30], [34]. The high isoelectric points are evocative of RNA-binding proteins although IQD proteins don't comprise currently known RNA-binding motifs. Fifty-seven soybean IQD proteins are localized to the nucleus, because of their high content of basic residues revealed by Wolf PSORT. TargetP analysis revealed that fifteen soybean IQD proteins are located in the mitochondria by identifying the presence of mitochondrial targeting peptide (mTP). The contradicting subcellular localization predictions is due to the different algorithm used by Wolf PSORT and TargetP. Most soybean IQD protein members are likely to function in the nucleus, as nucleus specific Ca^2+^-signatures are reported to generate in plant cells [55]–[57] and calmodulin and related Ca^2+^sensor proteins may play a regulatory role in nuclear processes such as transcription [58], [59]. Observably, *Arabidopsis IQD1* was reveraled to target to microtubules as well as the cell nucleus and nucleolus [32]. In vitro binding to single-stranded nucleic acids suggests *AtIQD1* and other IQD family members may control and fine-tune gene expression and protein sorting by facilitating cellular RNA localization [32].

### Phylogenetic analysis and evolution of IQD family genes

IQD proteins are an ancient family of CaM/CML binding proteins that originated during the early evolution of land plants, as IQD genes are present in *Physcomitrella patens*. ESTs corresponding to IQD proteins for angiosperm species (*Arabidopsis*, rice, etc.) and at least nine homologous sequences in the gymnosperm pine (*Pinus* ssp.) corresponding to IQD proteins were identified suggesting that the IQD gene family originated not later than the split of gymnosperms and angiosperms about 300 Myr ago [30]. We performed a genome-wide comparison of plant IQD members from monocots (rice and *Brachypodium distachyon*) and eudicots (soybean, *Arabidopsis* and tomato) to explore how the IQD gene family has evolved. The plant IQD members from monocots (rice and *Brachypodium distachyon*) and eudicots (soybean, Arabidopsis and tomato) appear to be more closely related to each other than to IQD genes of the same species in a different subfamily. This alternating distribution of monocots and eudicots in all subfamilies suggests that an ancestral set of IQD genes have existed before the dicot–monocot split (Figure 4, Table 4). The presence of five distinct subfamilies of IQD genes and the presence of both monocots and eudicots containing members in all five subfamilies indicate IQD genes have diversified before the monocot–eudicot split (Figure 4). These subfamilies include 23 pairs of orthologous genes, suggesting that orthologous genes may have originated from a common ancestor (Figure 4). About half of the orthologous genes (10 pairs; *BdIQD1*/*OsIQD8*:N, *BdIQD5*/*OsIQD3*:N, *BdIQD8*/*OsIQD10*:C, *BdIQD9*/*OsIQD23*:C, *BdIQD11*/*OsIQD19*:N, *BdIQD14*/*OsIQD28*:N, *BdIQD17*/*OsIQD24*:N *BdIQD18*/*OsIQD7*:N, *BdIQD20*/*OsIQD2*:N, *SlSUN13*/*GmIQD60*:N) have the same predicted subcellular localization suggesting that the encoded proteins may play similar roles in both species [30], [34]. A total of 87% (20 pairs) of orthologous gene pairs from rice and *Brachypodium distachyon* are distributed in all subfamilies. However, only two pairs of orthologous genes from dicotyledons (soybean and tomato) are from subfamily I and III. This difference may be due to the fact that both rice and *Brachypodium distachyon* are in the grass family and are therefore more closely related than *Arabidopsis*, soybean and tomato, which belong to Cruciferae, Solanaceae and Leguminosae, respectively. The number of soybean genes in each subfamily is greater than that of the other four species examined suggesting that IQD counterparts in soybean may have undergone gene expansion.

The duplication of individual genes, chromosomal segments or entire genomes has been a major force in the evolution of plant genome structure and content during the process of genome evolution [60], [61]. The soybean genome has undergone at least two round of duplication, resulting in the presence of significant features of remnants of a glycine-specific genome duplication that occurred ∼13 Mya and fainter remnants of older polyploidies prior to the divergence of the papilionoids (58–60 Mya) that occurred ∼58 Mya [41], [51]. Thus, 75% of soybean genes are present in multiple copies [41]. Among 67 soybean IQD genes, *GmIQD3*, -*32*, -*56* and -*60* were found as single copies on duplication blocks. These results suggest that segmental duplication has occurred as a continuous process and dynamic changes may have occurred in a chromosomal segment that contained two ancestral IQD genes, leading to corresponding sister gene loss [62]. One paralogs (*GmIQD11*/-*39*) shares 91.2% identity and similar exon/intron organization, but exists outside of any duplicated blocks. this pair might have been produced by retrotransposition. A high proportion (approximately 96%) of soybean IQD genes reside preferentially in duplicated segments, suggesting that segmental duplications have played a prominent role in the expansion of the soybean IQD gene family. The duplicated IQD genes in soybean have been preferentially retained at the high rate of 92.5% (62/67), which is distinctly higher than the retention rate (67.3%) of duplicated paralogs in the 1.1-gigabase sequence of the soybean (cv. Williams 82) genome, in which 31,264 genes exist as 15,632 paralog pairs (out of the 46,430 predicted high-confidence genes that were duplicated and retained after the 13-Mya tetraploidy event) [63]. The higher retention rate corroborates previous findings that genes involved in signal transduction are preferentially retained following duplications[64]. Our calculation of the duplication dates of the 31 paralogous pairs revealed that all of the segmental duplication events in the soybean IQD family occurred during the recent whole genome duplication event.

During evolution, eukaryotic genomes have retained genes on corresponding chromosomes (synteny) and in corresponding orders (collinearity) to various degrees. Synteny broadly refers to parallels in gene arrangement in dissimilar genomes. Collinearity, a specific form of synteny, requires genes to occur in largely corresponding orders along the chromosomes of respective genomes. According to the microsynteny analysis, microsynteny relationships among *AtIQD3*, *AtIQD4* or *SlSUN13* with other IQD III members in these three dicot genomes were not observed indicating that either these genes are ancient genes without detectable linkage to other IQD genes or that they were formed through complete transposition and loss of their primogenitors. In addition, three different duplicated chromosomal segments (harboring *AtIQD5*, *GmIQD32* and *GmIQD6*) that lost their sister IQD genes lack detectable microsynteny relationships to all other IQD III genes in the soybean and *Arabidopsis* genome, respectively. In the four IQD III gene clades, genes from soybean, tomato and *Arabidopsis* exhibit high levels of microsynteny, which indicates the IQD III genes existed before the divergence of the three dicotyledons genomes (soybean, tomato and *Arabidopsis*). Microsynteny was detected in most pairs, and alignment in clade 1–3 was discordant, suggesting that these genes may all be present in genome regions that were inverted, expanded or contracted after the divergence. Notably, most gene pairs in clade 4 have successive collinearity in order and the same orientation, which indicates high conservation among these IQD III gene-residing regions, with little rearrangement. The low (18.41%) synteny quality of IQD III genes from the three dicotyledon genomes (soybean, tomato and *Arabidopsis*) may have been due to the fact that these plants are not closely related; moreover, the gene density differs between *Arabidopsis* and the two other species. Significantly, the number of synteny blocks (31) within the soybean genome is much more than the number (3 or 4) of synteny blocks between tomato or *Arabidopsis* genomes, which suggests that soybean IQD III genes may have undergone large-scale duplication events and less rearrangement was followed (Figure 5 and Table S5b). The gene expansion pattern analysis of soybean paralogs indicates that most pairs were generated from a large-scale duplication, which supports the results of soybean gene duplication analysis, with the exception of *GmIQD41*/-*47*.

### Organ- or tissue-specific expression of IQD genes and expression of GmIQD III genes under MeJA stress treatment

Organ- or tissue-specific expression patterns have been observed for quite a few members of the IQD family. However, the functions of soybean IQD genes remain unclear. We therefore performed a thorough analysis of the RNA-Seq Atlas to investigate organ- or tissue-specific expression of IQD genes and qRT-PCR to examine the expression of GmIQD III members under MeJA stress treatment.

The tissue expression data deficiency of eight soybean IQD genes potentially indicated that these are pseudogenes or express only at specific developmental stages or under special conditions. 65.7% soybean IQD genes constitutively express in all of the seven tissues suggesting that GmIQDs may play roles at multiple developmental stages. Eight GmIQD proteins peak in only one tissue indicating that these tissue-specific calmodulin target proteins may be limited to discrete cells or organs to regulate various cellular activities.

Except for group C comprised of genes from GmIQD I, group A, B and D comprise genes from four subfamilies indicating these soybean IQD genes exhibit similar transcript abundance profiles but are relatively phylogenetically distinct. The analysis indicated that only some members within the same phylogenetic subgroup share a similar expression profile in soybean organs/tissues during development, excluding *GmIQD6* and *GmIQD26* belong to GmIQD IIb. For instance, *GmIQD4*, -*34*, -*38* and -*65* belong to GmIQD Ib clustered in group with high expression in flowers suggesting their potential roles in flower formation. While the other two GmIQD Ib members (*GmIQD8* and -*64*) were detected in nodules indicating they may involve in fixing atmospheric nitrogen.

Members possessing similar sequences are clustered in the same subfamilies, which may have similar expression patterns or functions. In IQD subgroup Ia, *Arabidopsis IQD22* is involved in the negative feedback regulation of GA-responsive DELLA genes [36]. Subgroup Ia members of *Arabidopsis IQD26* has higher expression level in parts with divided vigorous growth and microtubule organization of leaves, root and flowers [65]. Eight of the of the thirteen soybean IQD Ia members (*GmIQD3*, -*16*, -*22*,-*27*, -*29*, -*31* -*37* and -*66*) have high expression in young fleaves. And six of them (*GmIQD15*, -*16*, -*27*, -*31* -*37* and -*66*) have high expression in one-cm pod or pod-shell 10-DAF. *GmIQD22* have obviously higher expression at seed 21-DAF, the period of seed cell division (3, 4 weeks after flowering) [66]. These founding suggested that soybean IQD Ia members may function in transport of signaling molecules, nutrient transport and cell division.

Mapping and positional cloning of the *SUN* locus revealed that this member of the IQD II subfamily was generated by duplication of a 24.7-kb region carrying the tomato *IQD12* gene, a major gene involved in the control of fruit shape, particularly length, in tomato [35]. *SUN* is expressed the highest in hypocotyl and shoot apex. Overexpression of *SUN* causes root reduction when applied auxin and prostrate growth and twisted stems indicating that *SUN* can affect auxin transport or response [38]. *SlSUN1* shows slightly higher expression in the hypocotyl, flower at anthesis and fruit at 10 and 20 DPA [35]. *SlSUN12* and *SlSUN21* highly are expressed in the hypocotyls and root respectively [35]. *SlSUN17* evenly expressed in almost all tissues [35]. Soybean IQD II members showed the similar expression profile (*GmIQD11*: highest in flowers; *GmIQD33*, -*39* and -*40*: slight high in flowers and *GmIQD6* and -*26*: highest in roots) indicating that GmIQD II members may play similar role in plant development.

*Arabidopsis IQD1* from the IQD III subfamily modulates the expression of several glucosinolate (GS) pathway genes, resulting in the alteration of glucosinolate content and composition to promote resistance to herbivory. *Arabidopsis IQD1*, the first functionally characterized IQD gene, is expressed in vascular tissues of hypocotyls, leaves, stems, flowers and roots, as revealed by histochemical analysis. Expression pattern analysis of soybean IQD genes revealed that genes from the soybean IQD III subfamily were mainly expressed in young leaves, flowers and roots (Figure 6). Jasmonic acid(JA) treatment leads to elevating levels of specific glucosinolate in *Arabidopsis* [67], [68]. And overexpression of *AtIQD1* causes the accumulation of glucosinolates. However, *AtIQD1* expression is independent of JA, as steady-state *AtIQD1* mRNA expression levels are not appreciably altered when externally applied JA and are also unaffected in mutants defective in JA synthesis or signaling (JA -*jar1* and *fad3-2 fad7-2 fad8*) [31]. Indeed, *AtIQD1* increases resistance against herbivory by augmenting and fine-tuning glucosinolate accumulation [31]. Glucosinolates with important roles in plant defense and human nutrition are a small but diverse class of defense related secondary metabolites in cruciferous species such as *Brassica* crops and the *Arabidopsis thaliana* [69], [70]. Obviously, soybean doesn't synthesize glucosinolates. Base on the microsynteny analysis of IQDIII members, we auspiciously found there exsited highly conserved microsynteny relationship between *AtIQD1* and soybean IQDIII members. And combined with phylogenetic analysis of IQDIII members, we speculated soybean IQDIII members might have the similar biological function with *AtIQD1* in defenses to insect herbivory.

Therefore, we performed qRT-PCR of 24 soybean IQD III with MeJA treatment to detect whether soybean IQD III genes defense to insect herbivory. Compared to *AtIQD1*, soybean IQD III genes exhibited different responses with the MeJA treatment. The qRT-PCR results showed that 23 of the 24 soybean IQD III subfamily genes were upregulated by MeJA treatment, except *GmIQD21*, seven genes (*GmIQD28*, -*41*, -*45*, -*48*, -*54*, -*60* and -*67)* were strongly induced by MeJA with relative expression more than 10-fold. *GmIQD45* even accumulated the highest transcripts approaching 70-fold at 12 h (Figure 7). Based on these, we speculate that IQD III genes in soybean may involve in defense to insect herbivory by JA pathway.

Orthologs may have equivalent functions, as they originated from a single ancestral gene in the last common ancestor of the species. Two pairs of orthologous genes (*SlSUN2*2/*AtIQD6* and *SlSUN31*/*AtIQD5*) were found between tomato and *Arabidopsis* (Figure S2). Similar expression patterns of these two pairs in tomato and *Arabidopsis* have been reported; *SlSUN31* and *AtIQD5* are almost ubiquitously expressed, whereas *SlSUN22* and *AtIQD6* are highly expressed in young flower buds [30], [34], [35].

Duplicated genes may face three different fates: nonfunctionalization (one copy becomes silenced); neofunctionalization (one copy acquires a novel, beneficial function while the other copy retains the original function) or subfunctionalization (both copies become partially compromised by the accumulation of mutations) [45], [71]. Paralogs originating from duplication within one organism may have more divergent functions. In the current study, several pairs of paralogs showed similar expression patterns, which suggests that they may share a common or similar function. For example, *GmIQD10*/*GmIQD18* were highly expressed in young leaves, and their expression peaked at 4 h in response to MeJA (Figure 7). Several pairs of paralogs have different expression patterns, suggesting that they play diverse roles in soybean development. For example, *GmIQD21*/*GmIQD28* are mainly expressed in young leaves. Upon MeJA treatment, *GmIQD28* was most highly expressed at 4 h while its sister gene *GmIQD21* was downregulated at all time points.

In conclusion, IQD proteins play fundamental roles in various plant developmental processes. Therefore, the systematic analysis of the soybean IQD gene family performed in the current study provides an important reference for further characterization of the biological functions of these proteins.

## Materials and Methods

### Identification of IQD family genes in soybean

To identify IQD proteins in soybean, the *Glycine max* genome database (release 1.0, http://www.phytozome.net/soybean.php) was searched using Basic Local Alignment Search Tool algorithms (BLASTP), with the published *Arabidopsis* IQD protein sequences and their IQ67 domain used as initial query sequences. Redundant sequences were then removed manually, and the Hidden Markov Model of the Pfam (http://pfam.sanger.ac.uk/search) [72] and SMART (http://smart.embl-heidelberg.de/) [73] databases were used to confirm each candidate sequence as a member of the IQD family [74]. A total of 33 *Arabidopsis*, one moss (*Physcomitrella patens*) and 23 *Brachypodium* IQD protein sequences were downloaded from Phytozome v9.0 (http://www.phytozome.net/), and 34 tomato IQD protein sequences were retrieved from the tomato WGS chromosomes (2.40; SL2.40) (SGNhttp://solgenomics.net). Finally, 27 rice IQD protein sequences were obtained from the TAIR database (http://rice.plantbiology.msu.edu). Accession numbers of published IQD proteins for *Arabidopsis*, rice, tomato, *Brachypodium* and moss were listed in Table S8.

Soybean IQD gene information, including the number of amino acids, ORF lengths and chromosome locations, was obtained from the Phytozome database. Physicochemical parameters including the molecular weight (kDa) and isoelectric point (pI) of each gene product were calculated using compute pI/Mw tool from ExPASy (http://www.expasy.org/tools/) and parameter (resolution) was set to average [75]. Subcellular localization was predicted using the TargetP 1.1 (http://www.cbs.dtu.dk/services/TargetP/) server and WoLF PSORT (http://wolfpsort.org/).

### Multiple alignment and phylogenetic analysis of IQD family genes

Multiple sequence alignment of all predicted soybean IQD protein sequences was performed with Clustal X2.0 software using default parameters. Then, based on this alignment, phylogenetic trees were constructed using Clustal X2.0 with the Neighbor-Joining (NJ) method, and bootstrap analysis was conducted using 1,000 replicates [76]. An unrooted NJ tree of 184 the full-length IQD protein sequences from soybean, rice, *Arabidopsis*, tomato, *Brachypodium* was constructed using Clustal X2.0 with one moss IQD protein (Pp1s38230v6.1) as the outgroup. The GmIQD genes were classified according to their phylogenetic relationships with the corresponding *Arabidopsis* and rice IQD genes. For microsynteny analysis of IQD III genes across soybean, tomato and *Arabidopsis*, a phylogenetic tree was constructed using MEGA 5.0 with the NJ method and bootstrap analysis was conducted using 1,000 replicates.

### Genomic structure

Exon and intron structures of individual soybean IQD genes were deduced using GSDS (Gene structure display server; http://gsds.cbi.pku.edu.cn/) via alignment of the cDNAs with their corresponding genomic DNA sequences [77].

### Identification of conserved motifs and putative calmodulin-binding sites

Online MEME (Multiple Expectation Maximization for Motif Elicitation) (http://meme.sdsc.edu/meme4_4_0/intro.html) was performed to identify the conserved motif structures encoded by GmIQD genes. The parameters were as followings: number of repetitions - any, maximum number of motifs -10, and the optimum motif width was constrained to between 6 and 200 residues. In addition, each structural motif was annotated using the Pfam (http://pfam.sanger.ac.uk/search) and SMART (http://smart.embl-heidelberg.de/) tools. All IQD protein sequences were examined against the Calmodulin Target Database (http://calcium.uhnres.utoronto.ca/ctdb/ctdb/home.html) to predict putative calmodulin-binding sites.

### Chromosomal location and gene duplication

The chromosomal location image of GmIQD genes was generated by MapInspect (http://www.plantbreeding.wur.nl/uk/software_mapinspect.html) according to chromosomal position information provided in the Phytozome database. To identify tandem and segmental duplications, two genes in the same species located in the same clade of the phylogenetic tree were defined as coparalogs. The SoyBase browser (http://soybase.org/gb2/gbrowse/gmax1.01/) [78] was queried to detect the segmental duplication coordinates of the target genes. Coparalogs were deemed to result from segmental duplication if they were located on duplicated chromosomal blocks [79]. Paralogs were deemed to be tandem duplicated genes if two genes were separated by five or fewer genes in a 100-kb region [80]. The local alignment of two protein sequences was calculated using the Smith-Waterman algorithm (http://www.ebi.ac.uk/Tools/psa/).

### Calculation of Ka/Ks Values

Amino acid sequences of each paralog pair were first aligned using Clustal X2.0. Then, the multiple sequence alignments of proteins and the corresponding cDNA sequences were converted to codon alignments using PAL2NAL (http://www.bork.embl.de/pal2nal/) [81]. Finally, the resulting codon alignment was subjected to calculation of synonymous (Ks) and non-synonymous (Ka) substitution rates using the CODEML program of PAML [82].

Based on a rate of 6.1×10^−9^ substitutions per site per year, divergence time (T) was calculated using the Ks value with the formula: T = Ks/(2×6.1×10^−9^)×10^−6^ Mya [45].

### Microsynteny analysis

For microsynteny analysis, IQD III genes from soybean, tomato and *Arabidopsis* as the anchors were localized to specific target genomic regions. Then, all protein-coding sequences of 100 kb flanking each anchor point were compared by pairwise BLASTP analysis. The syntenic blocks used to construct synteny analysis maps of the IQD III genes were obtained from the Plant Genome Duplication Database (http://chibba.agtec.uga.edu/duplication/), a web service providing synteny information in terms of collinearity between chromosomes [83]–[85]. A synteny block is defined as a region where three or more conserved homologs are located within a 100-kb DNA stretch in both genomes. Two regions were considered to have originated from a large-scale duplication event when five or more protein-coding gene pairs flanking the anchor point were ligatured with the best non-self match (E-value<1e−10) [48]. The relative syntenic quality in a region was calculated from the sum of the total number of genes in both conserved gene regions (excluding retroelements and transposons and collapsing tandem duplications) [47].

### RNA-Seq atlas analysis

To acquire the tissue-specific transcript data, a list of 67 GmIQD gene names was entered to the RNA-Seq Atlas of *Glycine max* (http://soybase.org/soyseq/) [86]. The raw digital gene expression counts of the uniquely mappable reads were normalized using a slight variation of the reads/Kb/Million (RPKM) method and the normalized data was download from this database [86]. Hierarchical clustering analysis was conducted using clustering distance “correlation” (Pearson correlation) and the clustering method used “complete” (complete linkage method) in R [87]. A heat map was generated in R using the pheatmap function [87].

### Plant growth and treatments

Soybean (*Glycine max* L.) Williams 82 was used in this study. Seedlings were grown in a growth chamber under the following conditions: temperature, 30°C; photoperiod, 12 h/12 h; photon flux density, 80 µmolm^−2^ s^−1^ and relative humidity, 50% [88]. For expression pattern analysis of soybean IQD genes under stress, four-week-old seedlings were treated with 100 µm MeJA in the growth chamber [89]. Jasmonic acid methyl ester (MeJA) (Sigma, 95%) was diluted 1∶10 with 95% ethanol, followed by a further dilution with MilliQ water containing 0.1% Triton X-100, resulting in a final concentration of 100 µmol/L MeJA. Untreated seedlings were used as a control. Leaves of MeJA-treated plants were collected at 0, 1, 2, 4, 8 and 12 h. After collection, the samples were immediately frozen in liquid N_2_ and stored at −80°C for RNA extraction. Three biological replicates were conducted per sample.

### RNA extraction and qRT-PCR analysis

An RNAprep Pure Plant Kit (Tiangen) was used to isolate total RNA from each frozen sample. Possible contaminating genomic DNA was removed using DNaseI supplied in the kit. First-strand cDNA was synthesized from the RNA using a PrimeScript™ RT Master Mix Kit (TaKaRa) according to the manufacturer's instructions. Gene-specific primers for the 24 GmIQD genes were designed using Primer5.0 (Table S9). Primer specificity was first checked using the primer-BLAST tool available on the NCBI website. Subsequently, by performing analysis of melting curves and analysis of visualization of amplicon fragments, we found primers were gene-specific only when corresponding melting curves generated a single sharp peak and the primers demonstrated an electrophoresis pattern of a single amplicon with the correct predicted length. A housekeeping gene constitutively expressed in soybean, *CYP2* (cyclophilin) [46], [90]–[93], was used as a reference for normalization. The qRT-PCR analysis was conducted on an ABI 7500 Real-Time PCR system (Applied Biosystems). The reactions were performed in a 20 µl volume containing 10 µl 2×SYBR® *Premix Ex Taq*™ II (TaKaRa), 6.0 µl ddH_2_O, 0. 4 µl ROX Reference Dye II, 2.0 µl diluted cDNA and 0.8 µl of each gene-specific primer. The PCR conditions were as follows: Stage 1: 95°C for 30 s; stage 2: 40 cycles of 5 s at 95°C and 34 s at 60°C; stage 3: 95°C for 15 s, 60°C for 1 min, 95°C for 15 s. At stage 3, a melting curve was generated to estimate the specificity of the reactions. Three biological replicates were used per sample.

The relative expression levels were calculated as 2^−ΔΔCT^ [△C_T_  =  C_T, Target_ - C_T, *CYP2*_. △△CT = △C_T, treatment_ - △C_T, CK (0 h)_]. The relative expression levels (2^− ΔΔCT, CK (0 h)^) in the untreated control plants were normalized to 1 as described previously [46], [94], [95]. If an efficiency of amplification was less than 2, the result was proofread. Statistical analyses were conducted using GraphPad Prism 5.01 software [96].

## Supporting Information

Figure S1 **Amino acid sequence alignments of IQ67 domains in soybean IQD protein sequences.** The multiple alignment results indicate the highly conserved IQD domains among the 67 identified soybean IQD protein sequences. The positions of the conserved IQ calmodulin binding motifs are shown. Identical residues of proteins are marked with an asterisk. The consensus sequence at the bottom was constructed with greater than 50% conservation among the 67 soybean IQD proteins. Red arrow indicates the position of the conserved phase-0 intron, which divides codons 16 and 17 of the IQ67 domain.
(TIF)

Figure S2 **Phylogenetic tree of full-length IQD III proteins from soybean,** ***Arabidopsis*** **and tomato.** The tree was generated with MEGA 5.0 using the NJ method with 1,000 bootstrap replicates. Dicotyledon (soybean, tomato and *Arabidopsis*) IQD proteins are marked with colored dots. IQD III proteins from soybean, *Arabidopsis* and tomato were divided into four clades (1–4) presented by different color.
(TIF)

Table S1 **Detailed information about the 10 motifs in soybean IQD proteins.**
(XLS)

Table S2 **Recent synteny blocks of soybean and soybean (13 Mya) genomes containing IQD genes.**
(XLS)

Table S3 **Pairwise identities between paralogous pairs of IQD genes from soybean.**
(XLS)

Table S4 **The synteny blocks used to construct microsynteny map.**
(XLS)

Table S5 **Number of conserved gene pairs and synteny blocks and relative syntenic quality.**
(XLS)

Table S6 **Transcription of soybean IQD genes, as determined by RNA-seq analysis.**
(XLSX)

Table S7 **Animo acid content of 67 soybean IQD proteins.**
(XLS)

Table S8 **Accession numbers of IQDs for** ***Arabidopsis thaliana*** **, rice, tomato,** ***Brachypodium distachyon*** **and moss.**
(XLS)

Table S9 **List of primer sequences used for qRT-PCR analysis of the 24 soybean IQD III genes.**
(XLS)
