# Correction: The IQD Gene Family in Soybean: Structure, Phylogeny, Evolution and Expression

**DOI:** 10.1371/journal.pone.0119318

**Published:** 2015-03-23

**Authors:** The PLOS ONE Staff

In the eighth sentence of the second paragraph under the subheading “Chromosomal location and gene duplication” in the Results section, the phrase “three or fewer genes” should be written as “five or fewer genes”.

The correct sentence is: A pair of genes separated by five or fewer genes within a 100-kb region on a chromosome may have resulted from tandem duplication.

In the fourth sentence of the second paragraph under the subheading “Examination of soybean IQD gene expression by qRT-PCR” in the Results section, “GmIQD2” should be written as “GmIQD28”.

The correct sentence is: 12 genes (GmIQD28, -32, -41, -44, -45, -46, -48, -50, -53, -54, -35, -43, -60 and-67) exhibited major changes in expression (relative expression scales from 0 to 5 up 0 to 80).
