# Supplementary figures and images for: The IQD Gene Family in Soybean: Structure, Phylogeny, Evolution and Expression
Source: PLoS One. 2014 Oct 24;9(10):e110896. doi: 10.1371/journal.pone.0110896 (PMC4208818; doi:10.1371/journal.pone.0110896)

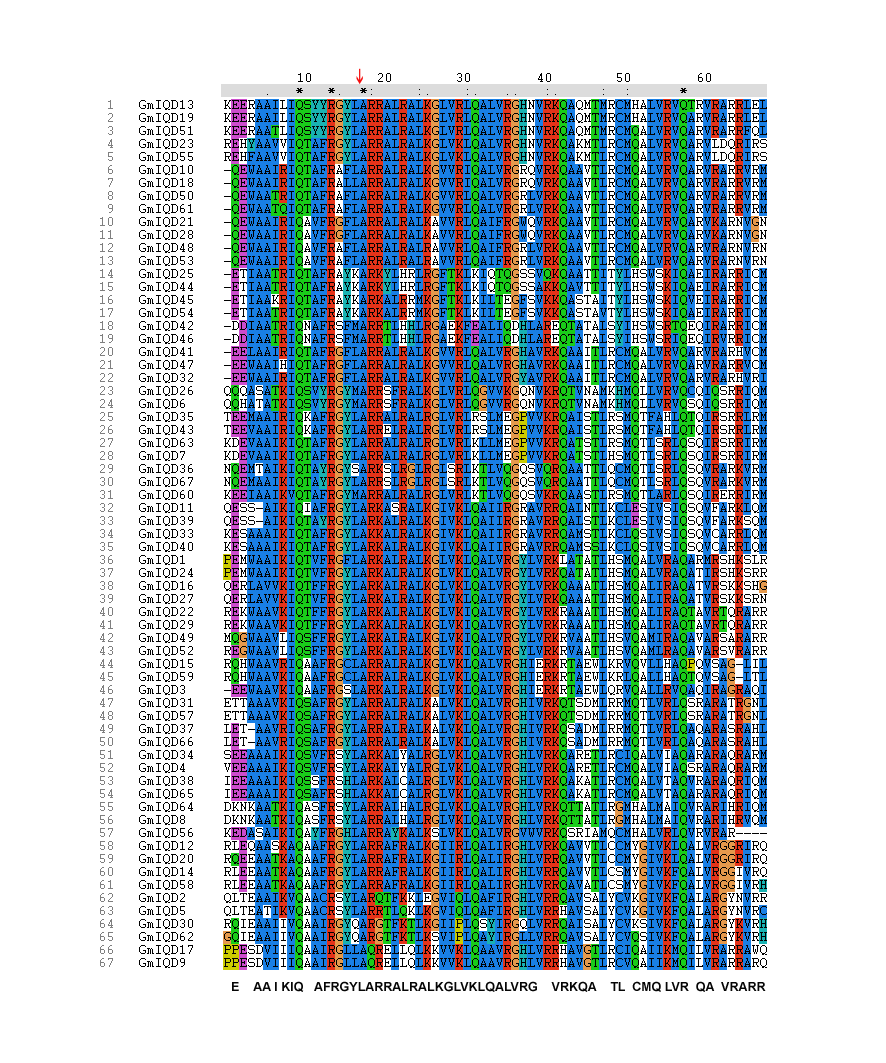

Supplement: Figure S1 — Amino acid sequence alignments of IQ67 domains in soybean IQD protein sequences. The multiple alignment results indicate the highly conserved IQD domains among the 67 identified soybean IQD protein sequences. The positions of the conserved IQ calmodulin binding motifs are shown. Identical residues of proteins are marked with an asterisk. The consensus sequence at the bottom was constructed with greater than 50% conservation among the 67 soybean IQD proteins. Red arrow indicates the position of the conserved phase-0 intron, which divides codons 16 and 17 of the IQ67 domain. (TIF) [file pone.0110896.s001.tif]

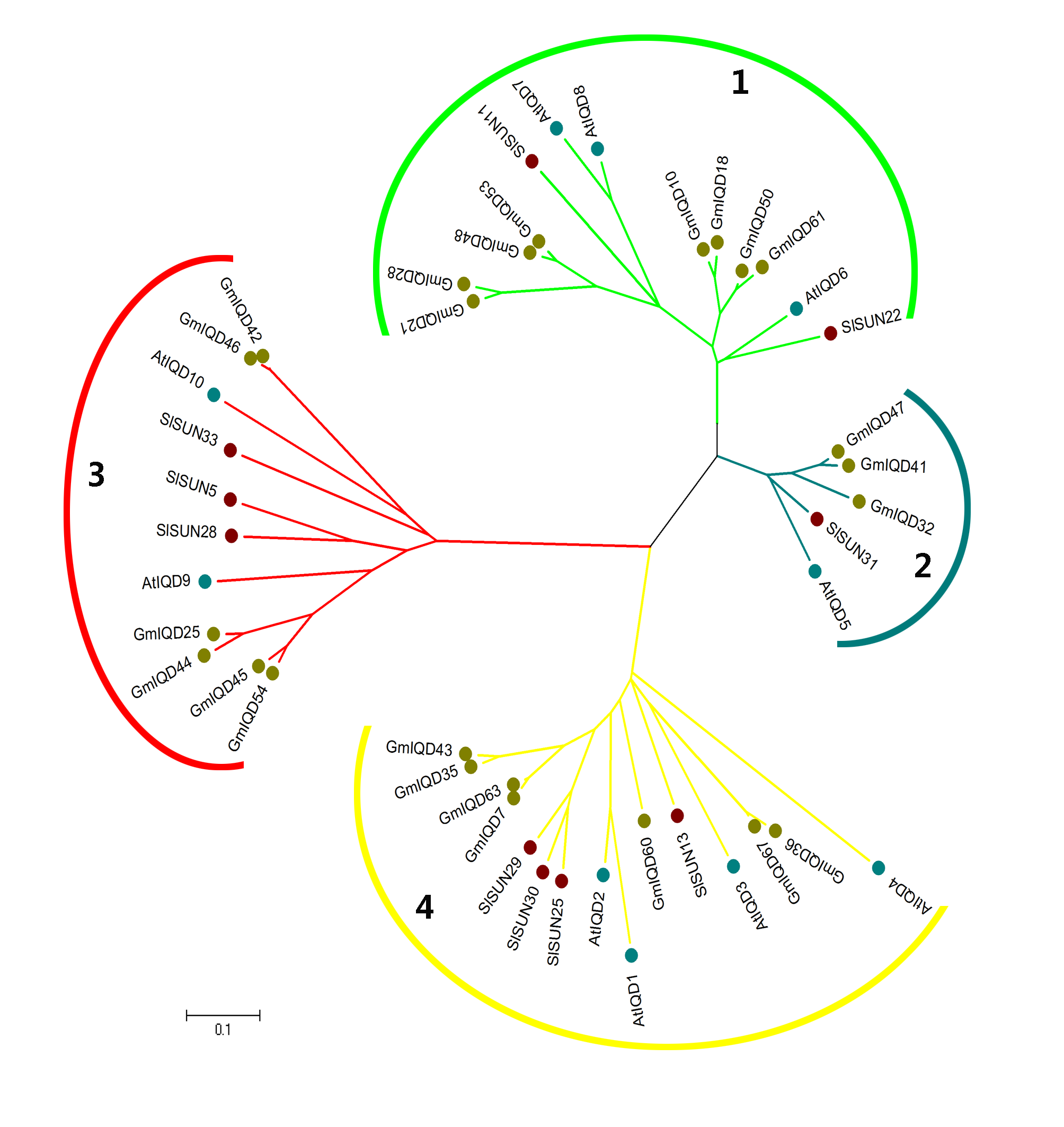

Supplement: Figure S2 — Phylogenetic tree of full-length IQD III proteins from soybean, Arabidopsis and tomato. The tree was generated with MEGA 5.0 using the NJ method with 1,000 bootstrap replicates. Dicotyledon (soybean, tomato and Arabidopsis) IQD proteins are marked with colored dots. IQD III proteins from soybean, Arabidopsis and tomato were divided into four clades (1–4) presented by different color. (TIF) [file pone.0110896.s002.tif]
